# MOTS-c-modified functional self-assembly peptide hydrogels enhance the activity of nucleus pulposus-derived mesenchymal stem cells of intervertebral disc degeneration

**DOI:** 10.1016/j.mtbio.2025.101872

**Published:** 2025-05-22

**Authors:** Yuan Lin, Ruo-yu Yang, Jie Li, Shan-zhong Shao, Xiang-qin Shi, Zhi-wei Huang, Shu-hai Zhang, Fu-jun Liu, Yin-shun Zhang, Sheng-quan Zhang, Su-mei Zhang, Tian-yong Wen, Hui Tao

**Affiliations:** a Department of Orthopedics, The First Affiliated Hospital of Anhui Medical University, Anhui Medical University, Hefei, 230022, Anhui Province, China; b Department of Orthopedics and Spine Surgery, The First Affiliated Hospital of Anhui Medical University, Hefei, 230022, Anhui Province, China; c Laboratory of Spinal and Spinal Cord Injury Regeneration and Repair, The First Affiliated Hospital of Anhui Medical University, Hefei, 230022, Anhui Province, China; d Department of Clinical Laboratory, the First Affiliated Hospital of Anhui Medical University, Hefei, 230022, Anhui Province, China; e Department of Operating Room, The First Affiliated Hospital of Anhui Medical University, Anhui Medical University, Hefei, 230022, Anhui Province, China; f Department of Radiology, The First Affiliated Hospital of Anhui Medical University, Anhui Medical University, Hefei, 230022, Anhui Province, China; g Department of Biochemistry and Molecular Biology, School of Basic Medicine of Anhui Medical University, Hefei, 230032, Anhui Province, China; h Senior Department of Orthopaedics, the Fourth Medical Centre, Chinese PLA General Hospital, Beijing, 100048, PR China

**Keywords:** Mitochondrial derived peptides, MOTS-c, Intervertebral disc degeneration, Nucleus pulposus mesenchymal stem cells, AMPK, Sirtuin 1, Self-assembling peptide RADA16-I, Nanofiber scaffolds, Oxidative stress

## Abstract

Intervertebral disc degeneration (IDD) is characterized by oxidative-stress driven progressive apoptosis and senescence of nucleus pulposus mesenchymal stem cells (NP-MSCs). MOTS-c, a 16-amino acid peptide encoded by the mitochondrial 12S rRNA open reading frame, has emerged as a key regulator of cellular metabolism, oxidative stress, and senescence. This study investigated the therapeutic potential of MOTS-c in countering tert-butyl hydroperoxide (TBHP)-induced oxidative damage in NP-MSCs, and we developed a novel biomaterial strategy for IDD treatment.

Key findings include:

**Mechanistic Protection:**

MOTS-c significantly attenuated TBHP-induced NP-MSC apoptosis (Annexin V+/PI + cells reduced by 48 %, p < 0.001), senescence (SA-β-gal + cells decreased by 52 %, p < 0.005), and ROS overproduction (35 % reduction, p < 0.0001) via activation of the AMPK/SIRT1 pathway. Pharmacological inhibition of SIRT1 abolished these protective effects, confirming pathway specificity.

**Functional Hydrogel Design:**

A sustained-release MOTS-c delivery system (RAD/RMOTS-c) was engineered by conjugating MOTS-c to the self-assembling RADA16-I peptide. The hydrogel exhibited a β-sheet-rich nanofibrous structure (fiber diameter: 362.6 nm), shear-thinning rheology (viscosity: 131–217 Pa s), and sustained peptide release over 7 days.

**In Vitro Efficacy:**

RAD/RMOTS-c enhanced NP-MSC viability (1.8-fold vs. control, p < 0.005) and extracellular matrix (ECM) synthesis, elevating collagen II/aggrecan expression (2.3-fold, p < 0.05) while suppressing collagen I (63 % reduction, p < 0.001).

In Vivo Therapeutic Validation: In a rat IDD model, RAD/RMOTS-c injection preserved disc height (DHI%: 82.4 vs. 58.7 in IDD group, p < 0.001), restored T2-weighted MRI signals (1.5-fold increase, p < 0.001), and reduced histological degeneration scores by 44 % compared to untreated controls (p < 0.001).

**Innovation and Impact:**

This work (1) demonstrates the association between MOTS-c's anti-degenerative effects and AMPK/SIRT1 signaling in NP-MSCs and (2) pioneers a peptide-hydrogel hybrid system that synergistically combines mitochondrial protection with structural support for disc regeneration. The findings can advance IDD therapy toward biology-driven, minimally invasive solutions, aligning with the paradigm of functional biomaterials for degenerative diseases.

## 1 Introduction

Intervertebral disc degeneration (IDD), driven by metabolic imbalance in nucleus pulposus (NP) cells, remains a major cause of age-related spinal disorders [1]. Current therapies—conservative, minimally invasive, or surgical—primarily alleviate symptoms without addressing structural repair or regeneration [2,3]. Emerging biological strategies, including mesenchymal stem cell (MSC) therapy and gene therapy, offer promising alternatives. Notably, endogenous NP-MSCs exhibit regenerative potential by differentiating into NP cells or inhibiting apoptosis, but their functionality declines with aging and IDD progression [4], underscoring the need to preserve NP-MSC activity for endogenous repair [[5], [6], [7], [8], [9], [10], [11], [12]].

Mitochondrial dysfunction critically influences stem cell homeostasis and aging [[13], [14], [15], [16]]. Mitochondria regulate redox balance, metabolite production, and stress responses, with mitochondrial-derived peptides (MDPs) emerging as key mediators of cellular adaptation [17]. Among these, the mitochondrial open reading frame of the 12S rRNA type-C (MOTS-c)—a 16-amino acid peptide encoded by the mitochondrial 12S ribosomal RNA region—modulates insulin sensitivity [18,19], oxidative stress, and senescence via AMPK signaling [18]. Mitochondria affect stem cells by regulating nuclear transcriptional programs [20,21] Recent studies highlight their role in rejuvenating aged stem cells, such as placental-derived MSCs, by restoring mitochondrial dynamics and activating AMPK [22]. Since the aging and apoptosis of NP-MSCs play an important role in IDD, we speculate that mitochondria and mitochondrial-derived peptides also play an important role in the aging and apoptosis of NP-MSCs. With advancements in MOTS-c research, we have further understood the role of MOTS-c in intracellular homeostasis, senescence, apoptosis, inflammation, and stress processes [[23], [24], [25]]. So far, MOTS-c has not been explored as an option in NP-MSC-mediated IDD therapy.

Local drug injection for the treatment of IDD often results in excessive local drug concentration or rapid drug catabolism, which ultimately renders the drug ineffective. To address challenges in localized IDD therapy, we engineered a sustained-release MOTS-c delivery system using RADA16-I self-assembling peptide hydrogel. RADA16-I forms a β-sheet scaffold with hydrophilic/hydrophobic interfaces [26], enabling covalent conjugation of functional peptides for controlled release. This platform not only seals annular defects but also provides a three dimensional (3D) microenvironment for NP-MSC adhesion and extracellular matrix deposition.

**Scheme 1:**
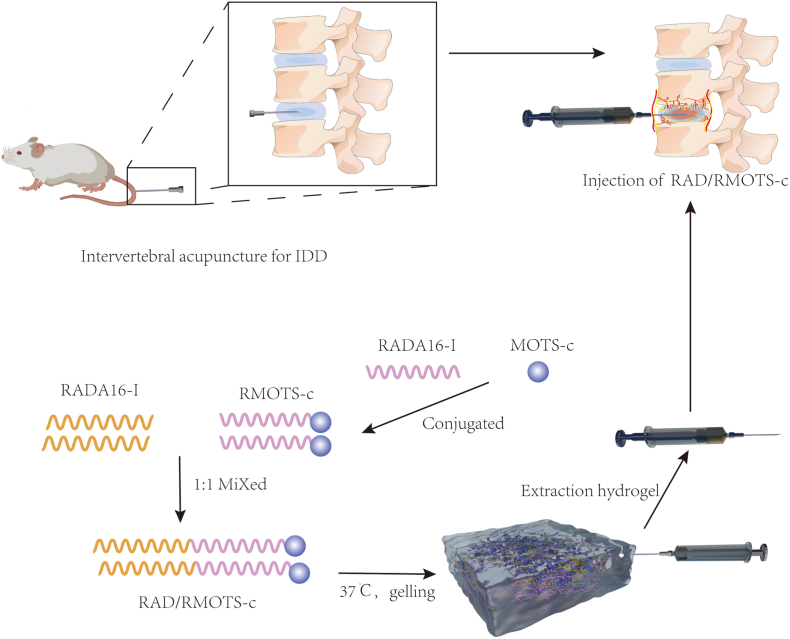
RAD/RMOTS-c hydrogel synthesis and its mechanisms in the treatment of intervertebral disc degeneration.

In this study, we demonstrate that MOTS-c-functionalized RADA16-I hydrogel (RAD/RMOTS-c) mitigates oxidative stress in NP-MSCs by activating the AMPK/SIRT1 axis, thereby preserving cell viability and function. Furthermore, in a rat IDD model induced by annular puncture, RAD/RMOTS-c hydrogel significantly attenuated disc degeneration, highlighting its translational potential. Our work integrates mitochondrial-targeted peptide therapy with biomaterial engineering, offering a novel strategy for IDD regeneration. The schematic diagram is shown in Scheme 1.

## 2 Materials and methods

### 2.1 Human tissue samples and ethics

Human NP tissues were obtained from patients with idiopathic scoliosis (IS) undergoing intervertebral disc surgery at the First Affiliated Hospital of Anhui Medical University. All procedures followed the Declaration of Helsinki and were approved by the hospital's ethics committee (approval no. [20200783]). Informed consent was obtained from all participants.(See Table 3)

### 2.2 Peptide preparation

The MOTS-c peptide (purity >90 %, Sangon Biotech, Shanghai, China) was synthesized using solid-phase methods. The lyophilized peptide was reconstituted in double-distilled water (ddH2O) at a stock concentration of 10 mg/mL, sterile-filtered (0.22 μm membrane), aliquoted into working concentrations (1 mM), and stored at −80 °C for long-term stability. The amino acid sequence is provided in Table 1.

**Table 1:** The peptide sequences of MOTS-c.

Name	sequence
MOTS-c	MRWQEMGYIFYPRKLR

RADA16-I and RMOTS-c Solutions.

The self-assembling peptides RADA16-I (Ac-RADARADARADARADA-CONH2) and RMOTS-c (Ac-(RADA)4-GG-MRWQEMGYIFYPRKLR-CONH2) were dissolved in sterile water at a concentration of 1 % (w/v). Solutions were filtered through 0.22 μm membranes and stored at 4 °C to maintain stability while preventing premature hydrogel formation.

RAD/RMOTS-c Hydrogel Fabrication.

To prepare the functionalized hydrogel, RADA16-I and RMOTS-c solutions were combined in a 1:1 volumetric ratio. This mixture was allowed to self-assemble under physiological pH conditions (7.4) to form a 3D scaffold optimized for subsequent cellular studies.

### 2.3 NP-MSC isolation and characterization

**Isolation:** NP tissues from patients with IS were minced, digested with 0.2 % collagenase II (Gibco, United States) (37 °C, 4 h), centrifuged (1500 rpm, 5 min), and cultured in F12 medium (Cyagen, United States) with 10 % FBS (HyClone, United States) and 1 % penicillin-streptomycin (Gibco, United States) [27]. Cells were sub-cultured at a ratio of 1:3 when they reached 80–90 % confluence. Cell growth and morphology of NP-MSCs were documented using a digital camera. The second passages of NP-MSCs were utilized for the subsequent experiments.

**Surface markers**: The effects of the surface markers CD34 (BD Pharmingen ^TM^, catalog no. 555824), CD73 (BD Pharmingen ^TM^, catalog no. 561254), CD45 (BD Pharmingen ^TM^, catalog no. 561866), CD90 (BD Pharmingen ^TM^, catalog no. 561969), CD105 (BD Pharmingen ^TM^, catalog no. 560839), and HLA-DR (BD Pharmingen ^TM^, catalog no. 559868) on MSCs were evaluated following the stem cell identification criteria outlined by the ISCT. Passage 2 NP-MSCs were analyzed using flow cytometry (FACSCalibur, BD, LSR II, Becton Dickinson USA) for CD34, CD45, CD73, CD90, CD105, and HLA-DR with FITC/PE/APC-conjugated antibodies (BD Pharmingen). The expression levels of individual surface markers were analyzed using the FLOW J software.

**Multilineage differentiation:** Osteogenic, adipogenic, and chondrogenic differentiation was induced using commercial kits, with differentiation confirmed using Alizarin Red (Procell, Wuhan, China, PD-014), Oil Red O (Procell, Wuhan, China, PD-016), and Alcian Blue staining (Procell, Wuhan, China, PD-015) respectively. Post-staining, the cells were washed again with PBS, and an inverted fluorescence microscope was utilized to observe the development of calcium nodules, lipid droplets, and sulfated proteoglycans. The staining outcomes were documented.

### 2.4 Immunofluorescence assay

Tissue Immunofluorescence: To evaluate MOTS-c expression patterns in NP tissues, frozen sections from normal (Pfirrmann grade ≤ II) and severely degenerated (grade IV) samples were incubated overnight at 4 °C with a primary antibody against MOTS-c (1:200 dilution). (See Table 2) Following PBS washes, Alexa Fluor-conjugated secondary antibody (1:200) was applied for 1 h at room temperature. Nuclei were counterstained with DAPI, and slides were mounted with an anti-fade reagent to preserve fluorescence signals. Imaging was performed using a wide-field fluorescence microscope (Nikon Eclipse Ti, Japan), with representative images captured for quantitative analysis.

Cellular Immunofluorescence: For mechanistic studies, NP-derived mesenchymal stem cells (NP-MSCs) were cultured under control conditions or exposed to oxidative stress (150 μM tert-butyl hydroperoxide, TBHP, 24 h). Cells were fixed with 4 % paraformaldehyde, permeabilized using 0.1 % Triton X-100, and blocked with 5 % BSA. Subsequently, immunostaining was performed using primary antibodies targeting MOTS-c, AMPK, or SIRT1 (1:200, overnight at 4 °C), followed by species-matched fluorescent secondary antibodies (1:200, 1 h). Fluorescence signals were visualized and analyzed using a confocal laser scanning microscope (Zeiss LSM 710, Germany).

### 2.5 In vitro experimental design

Oxidative stress model: NP-MSCs were pretreated with MOTS-c (100 μM, 24 h) ± EX527 (SIRT1 inhibitor, 10 μM, 2 h) before being exposed to Tert-Butyl Hydroperoxide (TBHP) (150 μM, 24 h) to induce oxidative stress. The experimental setup was categorized as follows: (1) Control group (blank); (2) TBHP group (TBHP 150 μM); (3) MOTS-c group (MOTS-c 100 μM + TBHP 150 μM); (4) SIRT1 inhibitor group (SIRT1 inhibitor 10 μM + MOTS-c 100 μM + TBHP 150 μM).

Hydrogel biocompatibility: NP-MSCs were encapsulated in RADA16-I or RAD/RMOTS-c hydrogels (1 % w/v) and cultured for 3 days. Cell viability was assessed using live/dead staining (calcein-AM/PI) and CCK-8 assays.

### 2.6 Cell counting Kit-8 assay

The cell viability of NP-MSCs was assessed using the cell counting kit-8 assay (C0038, Beyotime). NP-MSCs were plated in 96-well plates at a density of 8 × 10^3^ cells. After 24 h of incubation at 37 °C and 5 % CO_2_, the NP-MSCs adhered and grew in the plates. For the TBHP-induced oxidative stress injury, varying concentrations of TBHP (0,25, 50, 75, 100, 125, 150, 200, and 250 μM) were added to the culture medium and incubated for 24 h. To evaluate the effect of MOTS-c on cell proliferation capacity, MOTS-c at concentrations of 1, 2.5, 5, 10, 100, 200, 300, and 400 μM was added to the culture medium and incubated for 24 h. Growth curves for TBHP and MOTS-c were determined by incubating the normal, TBHP, and MOTS-c groups with respective drug concentrations for 1, 3, 5, and 7 days, changing the culture medium every 2 days. The protective effect of MOTS-c against TBHP damage was demonstrated by pre-incubating the wells with different MOTS-c concentrations (1, 2.5, 5, 10, 100 μM) for 24 h, subsequently adding or not adding 150 μM TBHP, and incubating for another 24 h. After the cells were treated as described above, the culture medium was replaced, a mixture of 10 μL of CCK-8 reagent and 100 μL of fresh medium was added to each well and incubated at 37 °C for 2 h, and the optical density (OD) values were measured at 450 nm using a spectrophotometer (M2, MOLECULAR DEVICE, USA). Each experiment was repeated thrice. Cell activity was calculated using the formula: Cytotoxicity (100 % control group) = [(Ae — Ab)/(Ac — Ab)], where Ae, Ab, and Ac represent the OD values of the treatment group, blank group, and control group, respectively.

### 2.7 NP-MSCs senescence-associated β-galactosidase staining

The senescence of NP-MSCs was evaluated using a Senescence-Associated β-Galactosidase (SA-β-Gal) staining kit (Beyotime, China) according to the manufacturer's instructions. Senescent cells exhibited elevated SA-β-Gal activity, resulting in blue staining. NP-MSCs were seeded in 6-well plates at a density of 4 × 10^4^ to 5 × 10^4^ cells/well and cultured at 37 °C with 5 % CO_2_. Following 24 h of cell adhesion, various treatments were administered according to the experimental design, and subsequent to PBS washing, cells were fixed with SA-β-fixative solution at room temperature for 15 min. Cells were then rinsed three times with PBS and subsequently incubated with SA-β-gal working solution at 37 °C overnight without CO_2_ (for a minimum of 12 h). Finally, staining of NP-MSCs was observed under a microscope and analyzed using the Image J software. Blue-stained cells indicated the presence of senescent NP-MSCs.

### 2.8 Detection of ROS

Intracellular levels of ROS in NP-MSCs were assessed using the fluorescent probe 2′,7′-dichlorofluorescein diacetate (DCFH-DA). NP-MSCs (5 × 10^5^ cells/well) were seeded in 12-well plates and cultured at 37 °C in 5 % CO_2_. After the cells were treated as described above, they were washed twice with PBS for 5 min each time, and then incubated with 10 μM DCFH-DA (diluted in serum-free medium) at 37 °C in the dark for 30 min according to the manufacturer's instructions. Subsequently, the cells were rinsed three times with serum-free medium to remove any unbound probes. Cell fluorescence images were promptly captured using a fluorescence microscope (Zeiss LSM 710, Germany) with excitation at 488 nm and emission at 525 nm.

### 2.9 Apoptosis related detection of NP-MSCs

#### 2.9.1 Apoptotic flow cytometry

The effect of MOTS-c on NP-MSCs apoptosis was analyzed using the Annexin V-FITC/PI apoptosis detection kit (KeyGen Biotech, China). Following varied treatments, NP-MSCs from each group were harvested through trypsin digestion and resuspended in 500 μl 1x binding buffer. Subsequently, 5 μL PI and 5 μL annexin-V were added to the cell suspension, followed by incubation in the dark at room temperature for 15–30 min. Flow cytometry (BD Biosciences, USA) was employed to quantify the proportion of cells expressing annexin V-FITC and PI markers. Apoptosis rates were determined by summing the percentages of early-apoptotic (Annexin V+/PI–) and late-apoptotic (Annexin V+/PI+) cells.

#### 2.9.2 In situ staining of apoptosis

Following treatment of NP-MSCs in various groups, the porous plates were centrifuged at 1000 g for 5 min. The cell culture medium was aspirated, washed thrice with PBS, and then centrifuged at 1000g for 5 min prior to PBS removal. 195 μL of Annexin V-FITC binding solution was added to the cells. Subsequently, 5 μL of Annexin V-FITC was added and gently mixed. The cells were incubated at room temperature (20–25 °C) in the dark for 10–20 min, followed by placement in an ice bath with aluminum foil for light protection. Annexin V-FITC fluoresces in green, while propidium iodide (PI) fluoresces in red when observed under a fluorescence microscope. Cell detection was performed within 1 h post-staining.

### 2.10 Real-time polymerase chain reaction (RT-PCR)

RT-PCR was employed to assess the gene expression levels of NP-MSCs across various treatment groups by detecting the expression of relevant genes. Total RNA was isolated using TRIZol reagent (Invitrogen, US, catalog no.15596-026) following the manufacturer's protocol, and RNA samples were treated with DNase/RNase-free water. The Nanodrop ND-1000 spectrophotometer (Nanodrop Technologies, USA) was utilized to assess the quality and quantity of RNA. cDNA was synthesized from total RNA using reverse transcription reagents (Takara, Japan). The mixture was incubated at 42 °C for 15 min, followed by incubation at 85 °C for 5 s, and then stored at −80 °C. The first-strand cDNA was amplified using PCR with the SYBR Premix Ex Taq PCR kit (Takara, Japan). The two-step amplification procedure included: 1) predenaturation at 95 °C for 30 s (1 cycle); 2) PCR reaction consisting of denaturation at 95 °C for 5 s and annealing/extension at 60 °C for 20 s (40 cycles); and 3) solution curve analysis at 65 °C for 15 s. Three replicate wells were established in each experimental group, and the average cycle threshold (Ct) value was determined. The relative gene expression levels were calculated using the 2^−ΔΔCt^ method. qPCR analysis was conducted using the LightCycler system (Roche, Switzerland). All genes were analyzed using RT-PCR, and mRNA levels were normalized to the housekeeping gene GAPDH. The Primer Premier 5.0 software was used to design the primer sequences, which are listed in Table 4.

**Table 2:** Pfirrmann grading table.

Grade	feature
Grade I	The structure of intervertebral discs presents a uniform white high signal, and the height of the intervertebral discs is normal
Grade II	The structure of intervertebral discs exhibits uneven white high signal intensity; The difference between annulus fibrosus and nucleus pulposus is quite obvious, with or without horizontal gray bands
Grade III	The signal intensity of intervertebral disc structure is uneven, with gray white signal intensity in the middle; The difference between annulus fibrosus and nucleus pulposus is not significant, and the height of the intervertebral disc is normal or slightly decreased
Grade IV	The signal of intervertebral disc structure is uneven, presenting as black low signal; The difference between the nucleus pulposus and annulus fibrosus disappears, and the height of the intervertebral disc is normal or moderately reduced
Grade V	The signal of intervertebral disc structure is uneven and appears as black low signal; The difference between the nucleus pulposus and annulus fibrosus disappears, and there is collapse of the intervertebral space

### 2.11 Western blot

Proteins from both the cytoplasm and nucleus of all cells were extracted using RIPA buffer supplemented with 1 % PMSF. Protein supernatants were collected via centrifugation at 4 °C for 20 min. Protein concentration was measured using the BCA protein quantification kit (Beyotime, China, catalog no. P0010). Equal amounts of protein samples (20 μg each) were collected from each group and subjected to 10 % sodium dodecyl sulfate-polyacrylamide gel electrophoresis (SDS-PAGE) for separation. Following separation, the gel was cut based on the molecular weight markers, and PVDF membranes were prepared accordingly. The proteins from the gel were transferred onto a polyvinylidene fluoride (PVDF) membrane using membrane transfer techniques. Following transfer, the PVDF membrane was sealed with 5 % skim milk in TBST-buffered saline (0.1 % Tween20) for 2 h at room temperature, then washed three times with TBST for 10 min each. Subsequently, the PVDF membrane was incubated overnight with primary antibodies at appropriate concentrations (1:5000–1:1000). Primary antibodies included anti-β-actin (1:1000, Abcam), anti-GAPDH (1:1000, ABclonal, Wuhan, China), anti-aggrecan (1:1000, Cell Signaling Technology Danvers, MA, USA), anti-Collagen II (1:1000, Cell Signaling Technology Danvers, MA, USA), anti-collagen I (1:1000, ABclonal, Wuhan, China), anti-SIRT1 (1:1000, ABclonal, Wuhan, China), anti-AMPK (1:1000, ABclonal, Wuhan, China), anti-p53 (1:1000, ABclonal, Wuhan, China), anti-P16 (1:500, ABclonal, Wuhan, China), anti-caspase-3 (1:500, ABclonal, Wuhan, China), anti-bcl-2 (1:1000, ABclonal, Wuhan, China), and anti-Bax (1:1000, ABclonal, Wuhan, China). Subsequently, the PVDF membrane was washed with TBST three times for 10 min each and then incubated with the corresponding rat or rabbit horseradish peroxidase-labeled secondary antibodies (1:10,000, ABclonal, Wuhan, China) for 1 h at room temperature with agitation. Following this, the membrane was washed three times for 10 min each with TBST. The membranes were visualized using an enhanced chemiluminescence system, and protein levels were quantified using the ImageJ software. GAPDH or β-actin served as internal controls.

### 2.12 Evaluation of circular dichroism

Solutions of self-assembled RADA16-I peptide, functional self-assembled RAD/RMOTS-c peptide, and RMOTS-c peptide were each diluted to 25 μM, transferred to quartz sample cells, and then inserted into a circular dichroism spectrometer. The path length was set to 0.5 cm, and the wavelength range was 190–260 nm, with a step size of 1 nm and bandwidth of 3 nm. The spectrum was acquired using a circular dichroism spectrophotometer. Each sample was measured thrice, and the final circular dichroism spectrum was obtained by averaging the three data points and subtracting the spectrum of distilled water.

### 2.13 Assessment of RADA16-I, RMOTS-c, and RAD/RMOTS-c using Atomic Force Microscopy

The self-assembled RADA16-I peptide solution, functional RMOTS-c peptide solution, and RAD/RMOTS-c peptide solution were diluted to a working concentration of 0.01 % (w/v) using Milli-Q water. Each diluted sample (5 μL) was deposited onto a freshly cleaved mica surface at room temperature. After 25–30 s, the mica surface was gently rinsed twice with 1000 μL of Milli-Q water to remove unattached peptides. Finally, the samples were air-dried and incubated at room temperature for 3–4 h. The microstructure of the samples was scanned using Atomic Force Microscopy (AFM) in air. AFM images were acquired using the tapping mode on a NanoWizard 4XP device (Bruker, Germany). All images were obtained with a setpoint of 0.3 V, Z length of 50 nm, Z speed of 29.17 μM/s, and pixel time of 4.286 ms. To visualize the nanostructure formed by the self-assembling peptides RADA16-I, RMOTS-c, and RAD/RMOTS-c, AFM images were acquired at scales of 1 × 1 μm^2^ with a resolution of 256 × 256 pixels.

### 2.14 Rheological analysis of self-assembled peptides

The self-assembled RADA16-I peptide solution and the functionalized self-assembled RAD/RMOTS-c peptide solution, both at a concentration of 1 % (w/v), underwent ultrasound treatment for 30 min. Subsequently, 100 μL of each polypeptide solution was absorbed and transferred to the parallel sample plate of the rheometer using a microsampler, where it was left to stand for 30 min. The fixture spacing was set at 300 μm, and the temperature was maintained at 37 °C. Three strain analysis modes were conducted: viscosity-shear rate relationship, strain scanning, and small amplitude oscillation.

### 2.15 Self-assembly assessment of RADA16-I and RAD/RMOTS-c using scanning electron microscope

The transwell chamber was placed into a 24-well culture plate containing 400 μL of medium per well. Subsequently, 100 μL each of self-assembled polypeptides RADA16-I and functionalized self-assembled polypeptide RAD/RMOTS-c were added to individual transwell chambers, followed by incubation at 37 °C for 1 h. Once gelation of the two solutions (RADA16-I and RAD/RMOTS-c) occurred separately, 400 μL of the medium containing cells was gently and slowly added to the surface. The self-assembled polypeptide hydrogel scaffolds, functionalized self-assembled polypeptide hydrogel scaffolds, and NP-MSCs were cultured for 3 days. The self-assembled polypeptide hydrogel cell scaffold and functionalized self-assembled polypeptide hydrogel cell scaffold were removed and fixed with a 2.5 % glutaraldehyde solution for 30 min. Following fixation, the scaffold was gradually dehydrated by immersion in ethanol solutions with concentrations of 30 %, 50 %, 70 %, 90 %, 95 %, and 100 %, respectively, for 10 min each. The scaffolds were dried for 3–4 h using the critical-point drying method (QUOROM, Britain). Once completely dried, they were sputter-coated with gold and examined under SEM. The microstructures and cell attachment of the two scaffolds were examined using the GeminiSEM 300 (Carl Zeiss, Germany) at magnifications ranging from 400 to 20,000 × and an acceleration voltage of 3 kV.

### 2.16 Cell survival rate assessment

NP-MSCs were labeled with calcein-AM (CAM) and propidium iodide (PI) to analyze their survival rate in various materials. CAM was prepared as a 10 μM/L working solution, and PI was similarly prepared to a concentration of 10 μM/L. NP-MSCs in various materials were washed thrice with PBS. Subsequently, the cells were fully covered with CAM working solution and incubated at room temperature in the dark for 20 min. The cells were then completely covered with PI working solution and incubated in the dark at room temperature for 10 min. Lastly, the incubation was terminated by removing the working solution, adding 500 μl of PBS, and observing the NP-MSCs double-stained with CAM and PI under a confocal fluorescence microscope.

### 2.17 Laboratory Animals and principles of care

Male Sprague-Dawley (SD) rats aged 8 weeks, obtained from Hangzhou Ziyuan Laboratory Animal Science and Technology Co., were used in this study. The rats were acclimatized to standard laboratory conditions for one week prior to the start of the study. In the laboratory, they were subjected to a 12-h light-dark cycle at a temperature of (25 ± 2 °C) and a relative humidity of 40–60 %, with access to adequate rodent chow and water. A total of 20 adult Sprague-Dawley rats were initially enrolled in this study. During the experimental timeline, four animals succumbed to perioperative complications, whereas two additional subjects were excluded due to unsuccessful modeling outcomes (failure to meet predefined MRI imaging validation criteria). Consequently, the final valid sample size comprised 14 rats. All experimental procedures adhered strictly to the National Institutes of Health Guide for the Care and Use of Laboratory Animals and the regulations set forth by the Animal Research Ethics Committee of the First Affiliated Hospital of Anhui Medical University.

### 2.18 IDD model induction and hydrogel material implantation

The IDD model in Sprague-Dawley (SD) rats was induced via annulus fibrosus (AF) puncture, as previously described [28]. Rats were anesthetized with intraperitoneal injection of 40 mg/kg of 1 % sodium pentobarbital, and anesthesia depth was confirmed using the tail pinch reflex test. Following sterilization with povidone iodine, Coccygeal (Co) 4–5, Co5-6, Co6-7, and Co7-8 were identified on sacral radiographs using a hypodermic needle. Co4-5 served as the control group, where only the epidermis was punctured without damaging the fibrous ring, whereas Co5-6 was designated as the IDD group, and puncture of the AF was conducted using an 18G needle inserted perpendicular to the caudal skin. To limit needle penetration to the AF, penetration depth was adjusted based on AF and NP dimensions, with an average depth of 5 mm established in preliminary experiments. Needle insertion depth was precisely controlled at 5 mm using a custom-made cover, ensuring puncture depth accuracy of 5 mm. The penetration depth was deemed adequate following confirmation of 5 mm depth and intraoperative X-ray localization observation parallel to the endplate and perpendicular to the skin. Upon reaching the correct position, the needle was rotated 360° and left in the intervertebral disc for 60 s. No sutures were necessary, as the procedure involved a simple puncture. The same procedure was repeated for the Co6-7 and Co7-8 discs. Rats were kept warm and closely monitored during recovery, ensuring access to food, water, and unrestricted exercise. One week later, MRI and X-ray imaging of the coccyx were conducted to assess disc degeneration. Upon confirming successful modeling, 5 μL of RADA16-I or RAD/RMOTS-c was injected into the NP of Co6-7 and Co7-8 using a microinjector puncture needle. Injection was delayed by approximately 5 min to minimize leakage.

The experimental groups were categorized as follows: Control (no treatment), IDD (puncture without treatment), RAD/RMOTS-c (injection of RAD/RMOTS-c hydrogel after puncture), and RADA16-I (injection of equal volume of RADA16).

### 2.19 Radiographic analysis

Orthopantomograms of the coccygeal bones of SD rats were acquired under anesthesia prior to modeling (0W), at 1 week post-modeling (1W), and at 4 weeks post-injection (5W) to evaluate alterations in disc height. Two independent imaging technologists, blinded to the study design, assessed the disc height index (DHI). Changes in DHI were expressed as DHI%, calculated using the formula: DHI% = (post-puncture DHI/pre-puncture DHI) × 100 %.

MRI scans of the rat coccyx were conducted prior to modeling (0W), at 1 week post-modeling(1W), and at 4 weeks post-injection (5W) to observe changes in intervertebral disc signal and structure. Sagittal T2-weighted images were acquired using the MAGNETOM Skyra 3.0 T superconducting MRI system with the following parameters: fast spin echo sequence, TR (time to repetition): 3500 ms, TE (time to echo): 107 ms, and slice thickness: 1.5 mm. Two blinded radiologists independently assessed MRI images using the intervertebral disc signal ratio.

### 2.20 Histological analyses of the caudal intervertebral disc

Caudal vertebrae of the rats were collected at the 4th week post-injection (5W). Samples were fixed in 4 % paraformaldehyde solution for 24–48 h. Decalcification was then conducted for 7–10 days using a slow bone tissue decalcification solution. Subsequently, the samples underwent dehydration using ethanol solutions of varying concentrations (75 %, 85 %, 95 %, and 100 %). Paraffin sections (6 μm thick) were prepared and stained with hematoxylin-eosin (HE) and Senka O (SO) staining solutions to assess the degree of IVDD in each group. The morphological structure of intervertebral discs, as well as the cell type and count within them, were assessed by three blinded observers and scored based on a grading scale obtained from previous studies. Histological grading of the disc degeneration is shown in Table 5.

**Table 3:** Tissue specimen information.

Number	Sex	Age	Operation	Pfirrmann grade	lumbar level
Case1	Female	55	UBE	Pfirrmann IV	L4-5
Case2	male	58	PELD	Pfirrmann IV	L3-4
Case3	male	42	PELD	Pfirrmann IV	L3-4
Case4	male	34	UBE	Pfirrmann IV	L4-5
Case5	female	16	ISO	Pfirrmann I	L1-2
Case6	male	14	ISO	Pfirrmann I	L2-3
Case7	male	15	ISO	Pfirrmann I	L2-3
Case8	female	14	ISO	Pfirrmann I	L3-4

Annotation:Percutaneous endoscopic lumbar discectomy, PELD; UnlateralBiportalEndoscopiy,UBE, ISO, Idiopathic scoliosis orthomorphia

**Table 4:** Primer sequences used for RT-PCR.

Gene	Forward Primer sequence (5' → 3′)	Reverse Primer sequence (5' → 3′)
Aggrecan Collagen II Collagen I Bax Bcl-2 Caspase-3 β-actin GAPDH P53 p16 SIRT1 AMPK	5′-ACGGCTTCTGGAGACAGGACTG-3′ 5′-GGAGCAGCAAGAGCAAGGAGAAG-3′ 5′-CCTGGAAAGAATGGAGATGATG-3′ 5′-ACT AAA GTG CCC GAG CTG AT -3′ 5′- GAG CAC CTG AAC CGG CAT CT -3′ 5′- GCA CAC GGG ACT TGG AAA GC -3′ 5′- CGTAAAGACCTCTATGCCAACA-3′ 5′-GAAGGTCGGAGTCAACGG-3′ 5′-ATGGAGGAGCCGCAGTCAG -3′ 5′-AGC CTT CGG CTG ACT GGC TGG-3′ 5′-AGATTTCAAGGCTGTTGGTTCC-3′ 5′-AGGAAGAATCCTGTGACAAGCAC-3′	5′-CTGGGATGCTGGTGCTGATGAC-3′ 5′-TCATCTGGACGTTGGCAGTGTTG -3′ 5′-ATCCAAACCACTGAAACCTCTG-3′ 5′- ATG GTC ACT GTC TGC CAT GT -3′ 5′-GAA ATC AAA CAG AGG TCG CA -3′ 5′- AGG AAG CCT GGA GCACAGAC -3′ 5′- CGGACTCATCGTACTCCTGCT-3′ 5′-GGAAGATGGTGATGGGATT-3′ 5′-TCAGTCTGAGTCAGGCCCTTC-3′ 5′-CTG CCC ATC ATG ACC TGG-3′ 5′-CAGCATCATCTTCCAAGCCATT-3′ 5′-CCGATCTCTGTGGAGTAGCAGT-3′

**Table 5:** Histological grading of the disc degeneration.

Cellularity and Morphology	Grade
Cellularity of the annulus fibrosus	1. Fibroblasts comprise more than 75 % of the cells 2. Neither fibroblasts nor chondrocytes comprise more than 75 % of the cells 3. Chondrocytes comprise more than 75 % of the cells
Morphology of the annulus fibrosus	1. Well-organized collagen lamellae without ruptured or serpentine fibers 2. Inward bulging, ruptured or serpentine fibers in less than one third of the annulus 3. Inward bulging, ruptured or serpentine fibers in more than one third of the annulus
Border between the annulus fibrosus and nucleus pulposus	1. Normal, without any interruption 2. Minimal interruption 3. Moderate or severe interruption
Cellularity of the nucleus pulposus	1. Normal cellularity with stellar-shaped nuclear cells evenly distributed throughout the nucleus 2. Slight decrease in the number of cells with some clustering 3. Moderate or severe decrease (>50 %) in the number of cells with all the remaining cells clustered and separated by dense areas of proteoglycans
Morphology of the nucleus pulposus	1. Round, comprising at least half of the disc area in mid-sagittal sections 2. Rounded or irregularly shaped, comprising one quarter to half of the disc area in mid-sagittal sections 3. Irregularly shaped, comprising less than one quarter of the disc area in mid-sagittal sections

The sections were deparaffinized, rehydrated, and microwaved in 0.01 mol/L sodium citrate for 15 min. Endogenous peroxidase activity was blocked with 3 % hydrogen peroxide for 10 min, whereas nonspecific binding sites were blocked with 5 % bovine serum albumin for 30 min. The sections were subsequently incubated overnight at 4 °C with primary antibodies (Collagen I, 1:200, ABclonal, Wuhan, China) and (Collagen II, 1:200, ABclonal, Wuhan, China). Finally, the sections were incubated with HRP-conjugated secondary antibodies (1:10000, Santa Cruz Biotechnology) and then counterstained with hematoxylin. All assays were conducted on a minimum of three sections from each sample.

### 2.21 Data analysis

Each measurement was performed in triplicate. Statistical analyses were conducted using SPSS23.0. Results are presented as mean ± standard deviation. Factorial design was analyzed using two-way analysis of variance (ANOVA), assessing main effects and group-time interactions, followed by one-way ANOVA for multiple group comparisons. The Student-Newman-Keuls test (for homogeneity of variance) or Tamhane test (for heterogeneity of variance) was employed for between-group comparisons. **A p-value < 0.05 was considered statistically significant.**

## 3 Results

### 3.1 Characterization of NP-MSCs

**Fig. 1:**
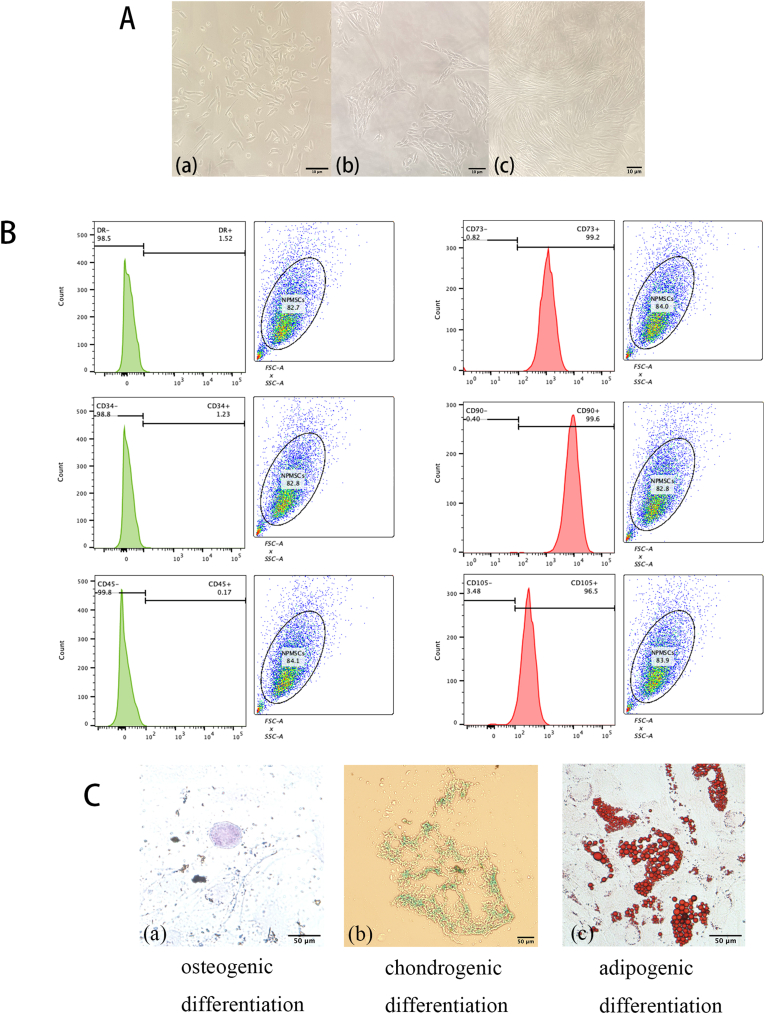
(A-a) NP-MSCs exhibited either a short rod or oval morphology after 3 days of primary culture. (A-b) Following 7–10 days of primary culture, NP-MSCs developed into partial clusters. (A-c) Upon 15 days of subculture, NP-MSCs displayed a uniform spindle-shaped morphology. Scale bar = 10 μm. (B) Flow cytometry analysis revealed the surface marker profile of NP-MSCs, indicating decreased expression of hematopoietic stem cell markers CD34, CD45, and HLA-DR, but elevated expression of mesenchymal stem cell markers CD73, CD90, and CD105. (C) NP-MSCs demonstrated positive staining for Alizarin Red, Oil Red O, and Alcian Blue following multilineage differentiation, indicating their osteogenic, adipogenic, and chondrogenic capabilities. Scale bar = 50 μm. (For interpretation of the references to colour in this figure legend, the reader is referred to the Web version of this article.)

Primary NP-MSCs isolated from human NP tissues exhibited short spindle morphology (P0, Fig. 1A–a) and formed clusters after 3–5 days (Fig. 1A and b). By passage two (P2), cells displayed uniform spindle-shaped morphology and rapid proliferation (Fig. 1A–c). Flow cytometry confirmed high expression of MSC markers (CD73, CD90, CD105) and low expression of hematopoietic markers (CD34, CD45, HLA-DR) (Fig. 1B), aligning with ISCT criteria [29,30]. NP-MSCs demonstrated trilineage differentiation potential, forming mineralized nodules (osteogenesis, Fig. 1C–a), sulfated proteoglycans (chondrogenesis, Fig. 1C–b), and lipid droplets (adipogenesis, Fig. 1C–c).

### 3.2 MOTS-c expression in degenerated IVD

**Fig. 2:**
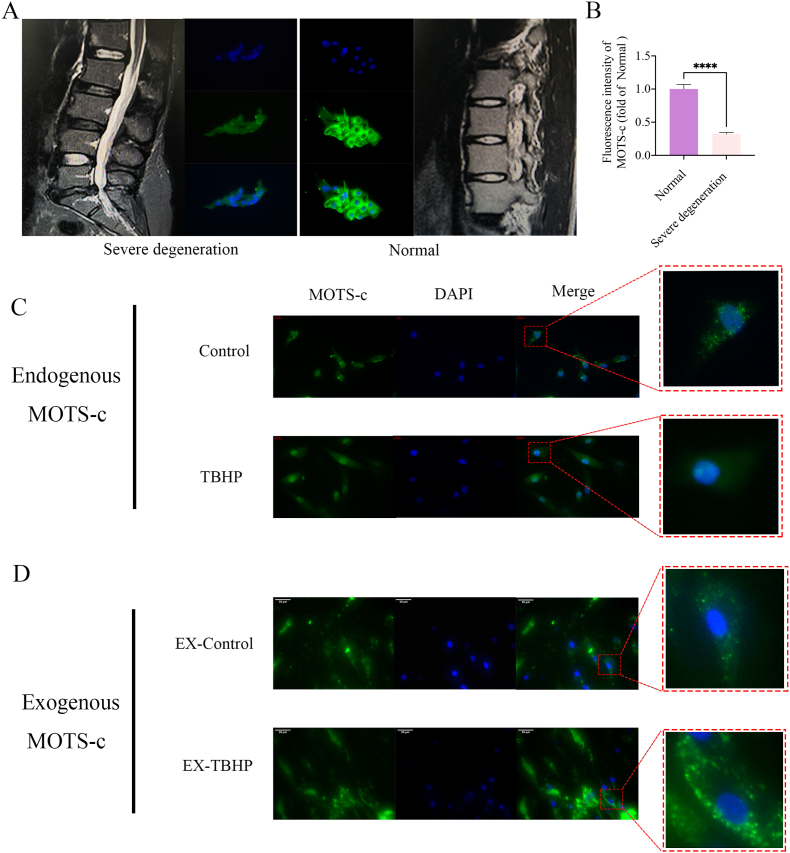
(A) The expression of MOTS-c is low in severely degenerated human nucleus pulposus tissue, while it is high in normal tissue. (B) A histogram shows the fluorescence intensity of MOTS-c in both severely degenerated and normal human nucleus pulposus tissue. (C–D) We examined the intracellular expression and localization of both endogenous and exogenous MOTS-C. The expression of endogenous MOTS-c in NP-MSCs was significantly higher in the control group compared to the TBHP group. In the control group, endogenous MOTS-c primarily localized in the cytoplasm, whereas its localization shifted significantly towards the nucleus in the TBHP group. After treatment with exogenous MOTS-c, it penetrated the cells and localized in the cytoplasm of NP-MSCs in both the control and TBHP groups. However, the quantity of exogenous MOTS-c invading the cells in the TBHP group was notably higher than in the control group (∗∗∗p < 0.001).

Immunofluorescence revealed significantly reduced MOTS-c expression in severely degenerated NP tissues (Pfirrmann grade III-IV) compared to normal/mildly degenerated tissues (grade ≤ II) (Fig. 2A and B, p < 0.0001), suggesting its role in IVD homeostasis.

### 3.3 MOTS-c localization under oxidative stress

Endogenous MOTS-c localized predominantly in the cytoplasm under normal conditions but translocated to the nucleus upon TBHP-induced oxidative stress (Fig. 2C). Exogenous FITC-labeled MOTS-c remained cytoplasmic even after TBHP exposure (Fig. 2D), indicating mitochondrial rather than nuclear targeting. In summary, the study confirmed the localization of MOTS-c in NP-MSCs and revealed that exogenous MOTS-c did not undergo significant nuclear translocation in TBHP-exposed NP-MSCs. Moreover, the cytoplasmic MOTS-c content decreased following oxidative stress stimulation, emphasizing the necessity of exogenous MOTS-c entry into the cytoplasm for replenishment in this experimental setup.

### 3.4 MOTS-c alleviates TBHP-induced decrease in NP-MSCs viability

**Fig. 3:**
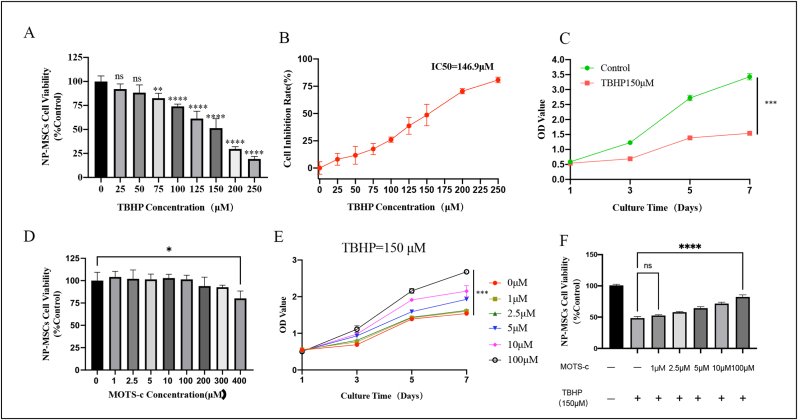
MOTS-c alleviates TBHP -induced decrease of NP-MSC viability. (A) Treatment with 0–250 μM TBHP for 24 h can appropriately inhibit the proliferative activity of NP-MSCs. (B) The IC50 diagram shows that 146.9 μM TBHP can inhibit the activity of NP-MSCs by half. (C) Culturing NP-MSCs with normal medium and 150 μM TBHP medium for 1–7 days resulted in significantly lower proliferation capacity due to oxidative stress injury induced by 150 μM TBHP compared to normal medium.(D) MOTS-c has no cytotoxic effect on the viability of NP-MSCs in the concentration range of 0 μM–100 μM (E) The proliferation capacity of NP-MSCs cultured with 0–100 μM MOTS-c increased gradually from day 1 to day 7, with the most significant promotion observed between days 3 and 5. (F) Different concentrations of MOTS-c significantly inhibited the cytotoxic effect of 150 μM TBHP on NP-MSCs, with the inhibitory effect of 100 μM MOTS-c being the greatest. All data are presented as mean ± standard deviation, based on a minimum of three independent experiments. (∗P < 0.05, ∗∗P < 0.001, ∗∗∗P < 0.005, ∗∗∗∗P < 0.001, ns, not significant).

Fig. 3A shows that TBHP at concentrations ranging from 0 to 250 μM decreased the viability of NP-MSCs after 24 h of incubation. Viability was 61.30 ± 7.71 % of the control at 125 μM TBHP (p < 0.0001) and 51.40 ± 9.79 % of the control at 150 μM TBHP (p < 0.0001). TBHP decreased NP-MSCs viability in a dose-dependent manner. The half-maximal inhibitory concentration (IC50) was chosen as the suitable TBHP concentration to model the oxidative damage of NP-MSCs, as determined from the cell growth inhibition curve. Cell inhibition rate = (Test OD value – Blank OD value)/(Control OD value – Blank OD value) × 100 %. The IC50 value was calculated as 146.9 μM based on the growth inhibition curve depicted in Fig. 3B. A concentration of 150 μM TBHP led to significant inhibition of cell viability. Therefore, this concentration was selected to induce oxidative injury damage in subsequent experiments.

To compare the time dependence of oxidative stress injury induced by TBHP, the growth curves of NP-MSCs cultured in normal medium and those exposed to 150 μM TBHP were analyzed over 1–7 days. The proliferation capacity of NP-MSCs was significantly reduced following oxidative stress injury induced by TBHP compared to the control medium as shown in Fig. 3C. The inhibitory effect of TBHP-induced oxidative stress injury was most pronounced at 24–48 h. Thus, the TBHP exposure duration was set at 24 h.

To assess the cytotoxic effects of MOTS-c on NP-MSCs, cells were treated with various concentrations of MOTS-c for 24 h. As depicted in Fig. 3D, cytotoxicity assays revealed no significant cytotoxic effects of MOTS-c on NP-MSCs at concentrations ranging from 0 to 100 μM for 24 h. 100 μM MOTS-c was selected as the maximum non-toxic concentration for subsequent experiments.

Dose and time response experiments were conducted to assess whether MOTS-c could enhance the cell survival of NP-MSCs exposed to 150 μM TBHP and to determine the optimal concentration and exposure duration of MOTS-c. MOTS-c was observed to protect NP-MSCs from TBHP-induced cell damage in a dose- and time-dependent manner. The protective effect of MOTS-c was most pronounced after 24–48 h, as depicted in Fig. 3E. The OD values of NP-MSCs exhibited a gradual increase with rising concentrations of MOTS-c, indicating that higher MOTS-c concentrations promoted the growth of NP-MSCs. MOTS-c exhibited maximum protective efficacy at a concentration of 100 μM (Fig. 3F, p < 0.0001).

In summary, NP-MSCs were exposed to 150 μM TBHP for 24 h and treated with 100 μM MOTS-c for 24 h in the subsequent experiments.

### 3.5 MOTS -c reduces TBHP-induced apoptosis of NP-MSCs

The anti-apoptotic effect of melatonin on NP-MSCs was evaluated in three ways.

**Fig. 4:**
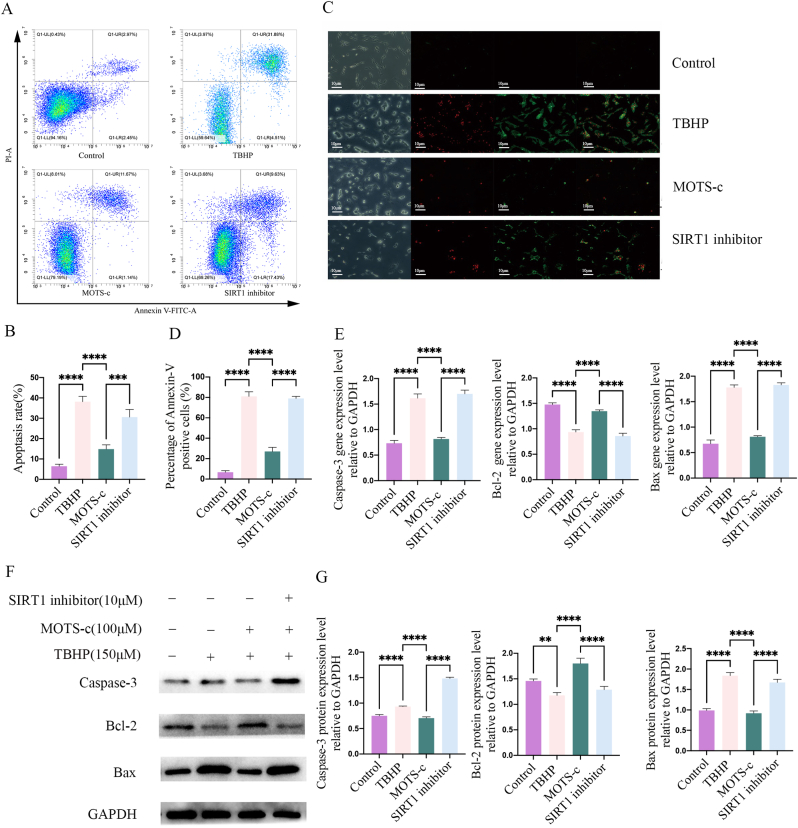
MOTS-c improved TBHP-induced NP-MSC apoptosis. The apoptosis rate of TBHP-treated NP-MSCs was significantly higher than that of the normal group, and MOTS-c partially decreased the apoptosis rate. However, the protective effect of MOTS-c could be significantly attenuated with the presence of SIRT1 inhibitor (A, B); Annexin-V PI assay result show that Annexin-V-positive cells increased significantly with TBHP treatment. Pre-treatment of MOTS-c significantly reduced Annexin-V-positive cells, whereas the protective effect of MOTS-c against TBHP-induced apoptosis was eliminated with the presence of SIRT1 inhibitor (C, D); MOTS-c significantly up-regulated the protein and mRNA expression of the anti-apoptotic molecule Bcl-2 and down-regulated the protein and mRNA expression of the pro-apoptotic molecules Bax and Caspase-3 in TBHP-induced NP-MSCs. However, the presence of the SIRT1 inhibitor eliminates the protective effect of MOTS-c on TBHP-induced apoptosis associated protein and mRNA expression (E, F and G). (∗,P < 0.05,∗∗,P < 0.01,∗∗∗,P < 0.005,∗∗∗∗,P < 0.001,ns, no significant).

First, the flow cytometry result indicated that the apoptosis rates of NP-MSCs were higher (p < 0.001) in the TBHP group compared to the Control group. However, MOTS-c decreased (p < 0.001) the TBHP-induced apoptosis rate of NP-MSCs, but this effect was reversed by pretreatment with SIRT1 inhibitor (Fig. 4A and B, p < 0.005).

Second, consistent with the flow cytometry results, the positive Annexin V staining rate, indicative of green fluorescence, significantly increased following TBHP treatment compared to the control group (p < 0.001). However, MOTS-c partially reversed this effect (p < 0.001). Furthermore, this protective effect of MOTS-c was weakened in the SIRT1 inhibitor group compared with the MOTS-c group (Fig. 4C and D, p < 0.001).

Third, to further assess the apoptosis-related effect of MOTS-c, the expression levels of apoptosis-related proteins were assessed. The expression of apoptosis-related proteins Caspase-3 and Bax (pro-apoptotic proteins) was increased (p < 0.001), whereas the expression of Bcl-2 (anti-apoptotic protein) was decreased (p < 0.01) in the TBHP group. In contrast, Caspase-3 and Bax were significantly decreased, and Bcl-2 was increased in the MOTS-c group (p < 0.001). Furthermore, SIRT1 inhibitor treatment reversed these effect of MOTS-c (p < 0.001). Consistent with the western blot (WB) result, the expression of anti-apoptotic gene (Bcl-2) was decreased, and pro-apoptotic gene (Caspase-3 and Bax) was increased in the TBHP group (p < 0.001). However, after the use of MOTS-c, the expression of Bcl-2 gene was significantly increased, and Caspase-3 and Bax genes were decreased in the MOTS-c group (p < 0.001). In addition, the expression of anti-apoptotic gene (Bcl-2) was downregulated, and pro-apoptotic gene (Caspase-3 and Bax) were upregulated in the SIRT1 inhibitor group (p < 0.001), which suggests that the SIRT1 signaling pathway plays an important role in inhibiting apoptosis (Fig. 4E–G, p < 0.001).

### 3.6 MOTS-c inhibits the senescence of NP-MSCs

**Fig. 5:**
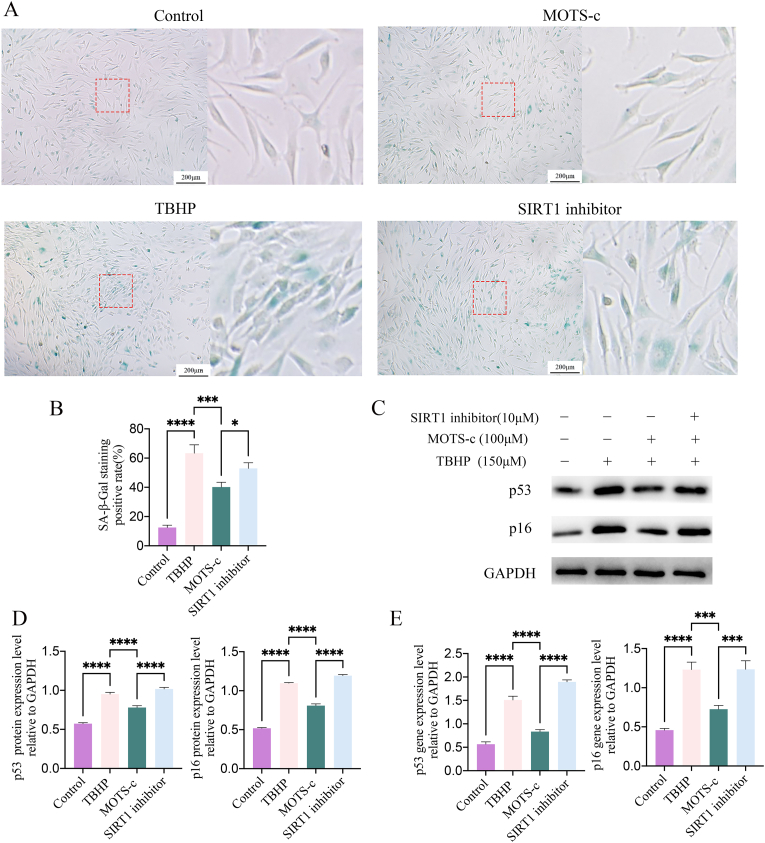
MOTS-c alleviated TBHP-induced senescence of NP-MSCs. (A–B) Data are expressed as percentage of blue staining by SA-β-Gal staining. (C–E) The SIRT1 inhibitor was able to eliminate the senescence of TBHP-induced NP-MSCs by MOTS-c. MOTS-c down-regulated the expression of senescence-associated p53 and p16 proteins and genes However, SIRT1 inhibitor eliminated the protection of MOTS-c against TBHP-induced senescence-associated p53 and p16 proteins and genes.(∗,P < 0.05,∗∗,P < 0.01,∗∗∗,P < 0.005,∗∗∗∗,P < 0.001,ns, no significant). (For interpretation of the references to colour in this figure legend, the reader is referred to the Web version of this article.)

The accumulation of SA-β-Gal indicates cellular senescence. SA-β-Gal staining showed that compared to the control group, the number of SA-β-Gal-positive senescent NP-MSCs in the TBHP group was significantly higher (p < 0.001), whereas the number of SA-β-Gal-positive NP-MSCs was decreased in the MOTS-c group (p < 0.005). Pretreatment with a SIRT1 inhibitor significantly attenuated the protective effect of MOTS-c, leading to an increase in the percentage of SA-β-gal-positive cells as shown in Fig. 5A and B (p < 0.05).

Western blot and RT-PCR were employed to assess the expression of cell senescence-related proteins (p53 and p16) in TBHP-induced NP-MSCs treated with MOTS-c. Senescence-associated proteins (p53, p16) were upregulated following TBHP treatment (p < 0.001), whereas MOTS-c treatment significantly attenuated this upregulation induced by TBHP (p < 0.001). Nonetheless, the SIRT1 inhibitor reversed the anti-senescence effect of MOTS-c, leading to a significant increase in the expression of p53 and p16 genes and proteins compared to the MOTS-c group as shown in Fig. 5C–E (p < 0.005). Thus, when the AMPK-SIRT1 pathway was inhibited by the SIRT1 inhibitor, the anti-aging effects of MOTS-c were significantly weakened. In summary, these findings collectively suggest that MOTS-c may exert a protective role against TBHP-induced oxidative stress injury in NP-MSCs, with the possible involvement of SIRT1 in conferring anti-senescence effects.

### 3.7 Effects of MOTS-c on ROS generated in TBHP-treated NP-MSCs

**Fig. 6:**
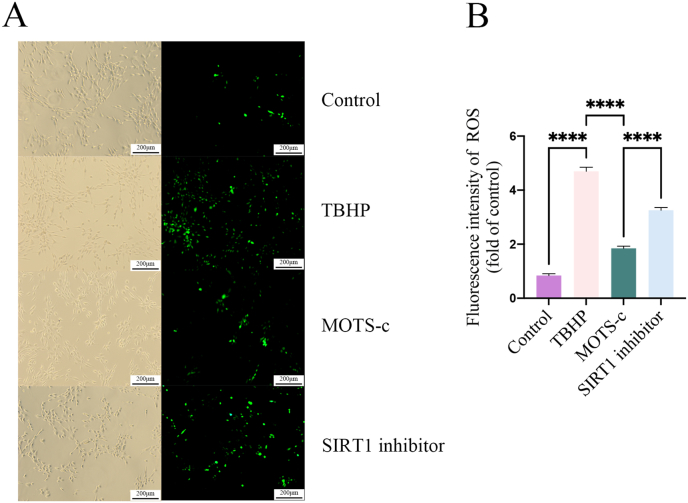
MOTS-c reduced TBHP-induced ROS expression. However, SIRT1 inhibitor eliminated the protection of MOTS-c against TBHP-induced ROS (∗∗∗∗,P < 0.001).

NP-MSCs in the TBHP group—subjected to oxidative stress—had higher ROS levels compared to the control group. Pretreatment with MOTS-c significantly reversed the TBHP-induced overproduction of ROS. The protective effect of MOTS-c on ROS levels was reduced by the SIRT1 inhibitor. These results are shown in Fig. 6A and B (p < 0.0001).

### 3.8 MOTS-c regulates the expression of ECM-related genes and proteins

**Fig. 7:**
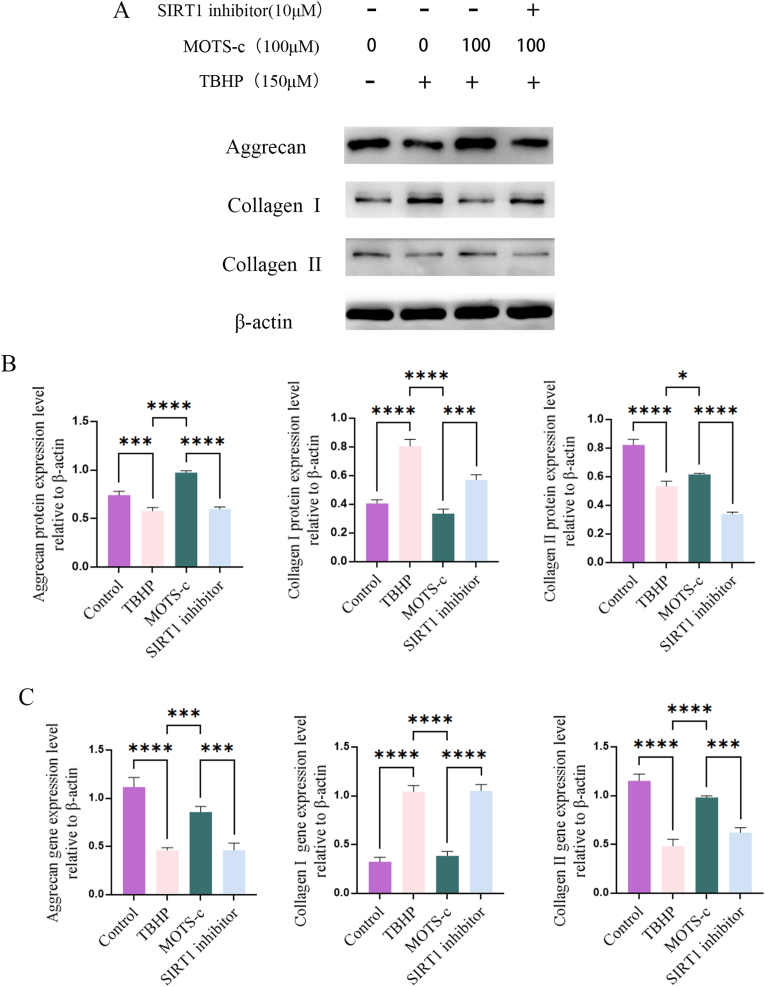
MOTS-c up-regulated the expression of Aggrecan and Collagen II proteins and genes and down-regulated Collagen I protein and genes in NP-MSCs. However, SIRT1 inhibitor attenuated the protective effect of MOTS-c on NP-MSCs.(∗,P < 0.05,∗∗,P < 0.01,∗∗∗,P < 0.005,∗∗∗∗,P < 0.001,ns, no significant).

The expression of ECM-related proteins in NP-MSCs was also analyzed. We found that TBHP treatment significantly inhibited the expression of collagen II and aggrecan and promoted the expression of collagen I (Fig. 7A—7C, p < 0.005). MOTS-c pretreatment showed a protective effect on NP-MSCs; the expression levels of collagen II and aggrecan were significantly enhanced, whereas the expression of collagen I was significantly weakened. Furthermore, the SIRT1 inhibitor pretreatment reversed this protective effect.

### 3.9 Activity of the AMPK-SIRT1 pathway in the protective effect of MOTS-c

**Fig. 8:**
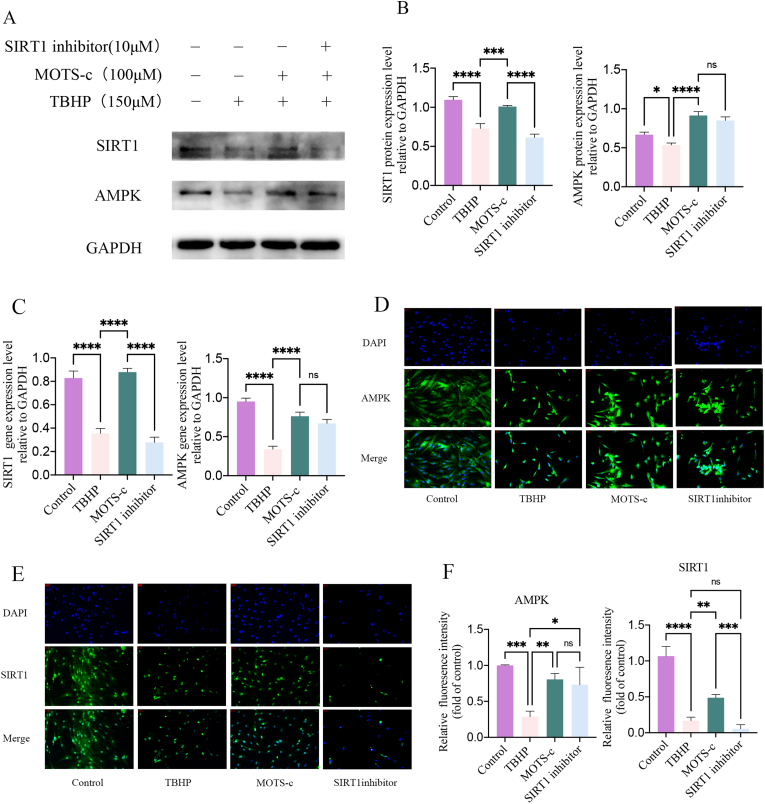
(A–C) Expression of AMPK-SIRT1 signaling pathway protein and gene in NP-MSCs against oxidative stress under different treatment conditions. (D–F) Intracellular immunofluorescence expression of AMPK and SIRT1 in different treatment groups. (∗,P < 0.05,∗∗,P < 0.01,∗∗∗,P < 0.005,∗∗∗∗,P < 0.001,ns, no significant).

Further investigations were carried out to mechanistically understand the role of MOTS-c in AMPK-SIRT1 interaction. The expression of the AMPK/SIRT1 signaling pathway-related proteins AMPK and SIRT1 were detected using RT-PCR and western blotting. As showed in Fig. 8A–C, compared with the control group, the gene and protein expression of AMPK and SIRT1 were significantly decreased in the TBHP group (p < 0.001). Pretreatment with MOTS-c increased the gene and protein expressions of AMPK and SIRT1 protein compared with the TBHP-treated group (p < 0.005). However, the addition of SIRT1 inhibitor significantly decreased SIRT1 gene and protein expression (p < 0.001). The expression levels of AMPK did not significantly change after the SIRT1 inhibitor treatment. These results indicate that the AMPK/SIRT1 signaling pathway is involved in alleviating TBHP-induced apoptosis, senescence, and oxidative stress in NP-MSCs, with AMPK likely acting upstream of SIRT1. Activation of AMPK was associated with increased SIRT1 protein expression.

The findings were also supported by AMPK and SIRT1 immunofluorescence staining. The results in Fig. 8D and E indicate a significant decrease in the fluorescence expression level of AMPK and SIRT1 in NP-MSCs after TBHP treatment, consistent with the western blot analysis. Treatment of NP-MSCs with MOTS-c led to a significant increase in the fluorescence levels of AMPK and SIRT1 (Fig. 8F, p < 0.05). The AMPK fluorescence expression levels remained similar between the SIRT1 inhibitor group and the MOTS-c group, with no significant difference noted. However, the SIRT1 fluorescence expression level was significantly decreased in comparison with the MOTS-c group. This indicates that the use of SIRT1 inhibitor could effectively inhibit SIRT1 expression.

### 3.10 RADA16-I and RAD/RMOTS-c form hydrogel-like materials

**Fig. 9:**
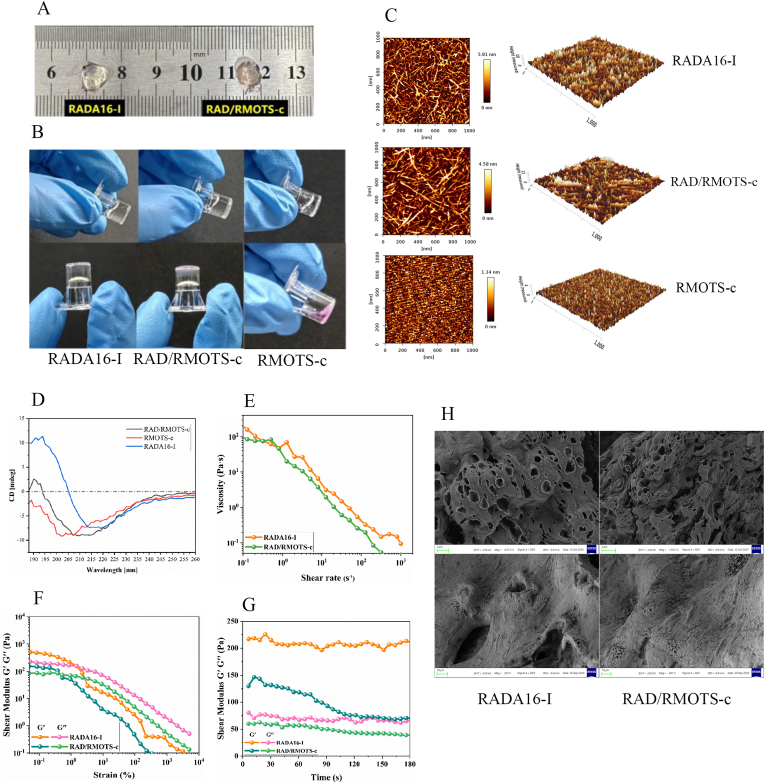
(A) Appearance and Hydrogel Formation of RADA16-I and RAD/RMOTS-c. Both RADA16-I and RAD/RMOTS-c are capable of forming hydrogel-like materials in vitro at 37 °C. (B) Verification Diagram of Glue Formation by RADA16-I and RAD/RMOTS-c. (C) Atomic Force Microscope Images and Three-Dimensional Structure Diagrams. Nanofibers can be formed in self-assembled polypeptide solutions of both RADA16-I and RAD/RMOTS-c. However, RMOTS-c cannot form nanofiber structures. (D) Circular Dichroism Spectra of RADA16-I, RMOTS-c, and RAD/RMOTS-c Materials. (E) Shear Rate-Viscosity Scanning Results of Polypeptide Hydrogel Scaffolds of RADA16-I and RAD/RMOTS-c. (F) Storage Modulus Scanning Results of Small Amplitude Oscillation Post-Formation of Polypeptide Hydrogel Scaffolds of RADA16-I and RAD/RMOTS-c. (G) Time Modulus Scanning Results of Small Amplitude Oscillation in Polypeptide Hydrogel Scaffolds of RADA16-I and RAD/RMOTS-c. (H) Microstructural Characteristics of RADA16-I and RAD/RMOTS-c Under Scanning Electron Microscopy.

MOTS-c was conjugated to the C-terminal of RADA16-I through solid-phase synthesis, resulting in the formation of the functional self-assembling polypeptide RMOTS-c. RMOTS-c and RADA16-I were mixed in a 1:1 ratio, resulting in the formation of a translucent viscous gel material at 37 °C in vitro. The appearance of the hydrogel formed by RADA16-I and R/RMOTS-c is depicted in Fig. 9A. No noticeable difference in appearance was observed between the two, and the gel can maintain stability without collapsing at normal temperature. As depicted in Fig. 9B, when the hydrogel chamber was tilted or inverted, RADA16-I and RAD/RMOTS-c remained stable without tilting, whereas the inability of RMOTS-c to form a gel led to tilting or flowing out of the chamber.

### 3.11 Atomic Force Microscopy

The nanostructures of RADA16-I, RAD/RMOTS-c, and RMOTS-c were observed using AFM (Tapping Mode) at a scale of 1 × 1 μm^2^ in this study. Fig. 9C depicts dense nanofibers formed by RADA16-I and RAD/RMOTS-c, which is consistent with a previous study. RMOTS-c did not form nanostructures. These results confirm the excellent self-assembling ability of peptides RADA16-I and RAD/RMOTS-c to form nanofibers and higher-order nanofiber scaffolds. Quantitative analysis revealed that RADA16-I formed shorter nanofibers (136.7 ± 18.2 nm length), whereas RAD/RMOTS-c self-assembled into elongated fibers (362.6 ± 41.5 nm length) (p < 0.001; n = 50 fibers/group). The length of nanofibers formed by RAD/RMOTS-c was longer than that formed by RADA16-I.

### 3.12 Analysis of results of circular dichroism

The circular dichroism (CD) spectrum of proteins is contingent upon their secondary structure. Changes in the secondary structure led to corresponding alterations in the CD spectra. The β-fold structure is characterized by a negative peak at 217–218 nm and a prominent positive peak at 195–200 nm. In this experiment, we observed that the CD spectrum of peptides, measured between 160 and 260 nm, indicated pronounced β-folding in RADA16-I. Additionally, the mixture of RMOTS-c with RADA16-I in equal proportions yielded a characteristic β-folding curve. The RMOTS-c material exhibits solely a negative peak at a wavelength of 195–200 nm, suggesting the formation of an irregular, coiled secondary structure. The CD spectra of the three materials are illustrated in Fig. 9D.

### 3.13 Analysis of rheological results

Following self-assembly of the two peptides RADA16-I and RAD/RMOTS-c into gels at 37 °C, the viscosity of the hydrogels was assayed. In this experiment, we observed that at a shear rate of 0 S^−1^, both peptide hydrogels exhibited high viscosity, maintaining stable morphology post gel formation. The viscosity of the two peptides post gel formation was found to be dependent on shear rate, as depicted in the viscosity-shear rate diagram. The viscosity measurements indicated that both peptide gels, RADA16-I and RAD/RMOTS-c, exhibit similar shear thinning behavior, with viscosity linearly related to shear rate in logarithmic scales, consistent with expectations, suggesting non-Newtonian fluid characteristics. Moreover, the viscosities of approximately 217 Pa s and 131 Pa s for the respective gels ensured post-gelation stability and effectively prevented potential shape collapse, as shown in Fig. 9E.

The yield property of a hydrogel is a critical parameter reflecting its long-term stability. Modulus, which describes a substance's elasticity (rigidity) by its ability to resist deformation and store energy, is a fundamental physical quantity. The yield stress needed to initiate flow is determined by the interplay between the storage (G′) and loss (G″) moduli. Fig. 9F shows that at small strains, the storage modulus exceeded the loss modulus for both gels, suggesting elastic and stable networks. However, as shear strain increased beyond a threshold, both G′ and G″ decreased markedly, indicating network disruption. Additionally, both gels displayed distinct plateaus in yield stress and modulus values, with G′ surpassing G". These findings illustrate the stability and pseudo-solid behavior of hydrogels, which contribute to their stability maintenance. Strain scanning results indicate that under lower stress conditions, both hydrogel materials offer cellular support, preventing cell collapse and deformation, thus averting extrusion damage. Under higher stress, these materials can supply a fluidic environment, promoting gradual peptide dissolution and release.

The results of small-amplitude oscillation time scans are depicted in Fig. 9F. After incubating the two hydrogel materials together in the incubator at 37 °C for 1 h, oscillation time-modulus scans were conducted at a fixed angular velocity of 1 rad/s. Ultimately, we observed that the reserve modulus of both hydrogel materials consistently surpassed the loss modulus, indicating the ability to form gels and maintain stability. Because the reserve and loss moduli of RAD/RMOTS-c were lower than those of RADA16-I, this suggests that the stability after gel formation is marginally inferior to that of RADA16-I.

### 3.14 Scanning electron microscope imaging

RADA16-I and RAD/RMOTS-c were incorporated into hydrogel scaffolds in vitro, and their ultrastructure were examined using SEM. As shown in Fig. 9H, the results demonstrated that all polypeptides were capable of forming numerous nanofibers, which interwove to create staggered and well-organized porous scaffolds in vitro. The diameter of the nanofiber pores was approximately 1–2 μm. In both hydrogel scaffolds, NP-MSCs adhered tightly to the nanofibers, whereas on the surface of the gel, they exhibited a fusiform shape.

### 3.15 Cytotoxicity assay

**Fig. 10:**
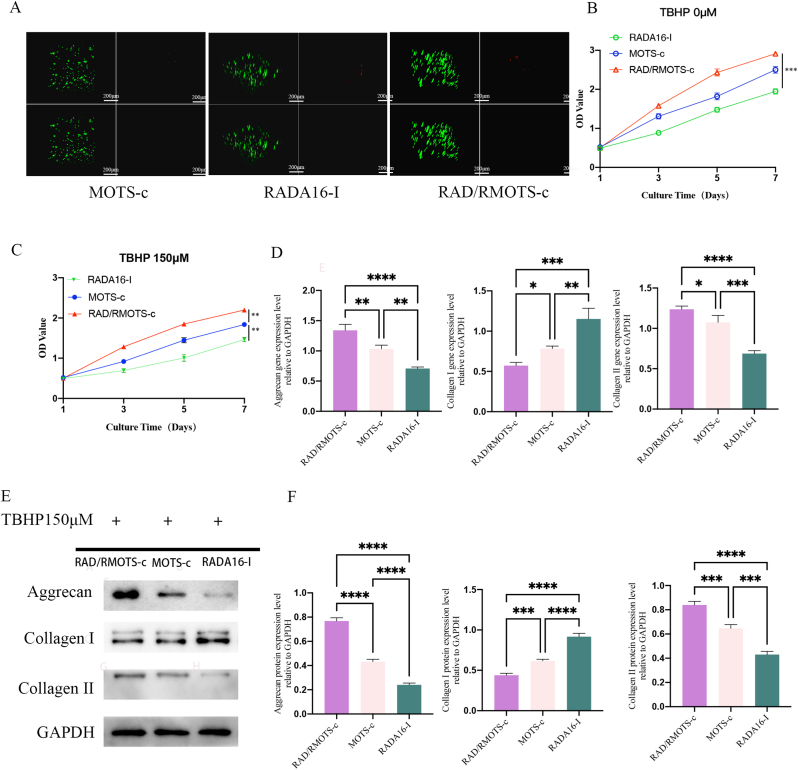
(A) Cell viability was assessed using live-dead staining after incubation in three different environments: MOTS-c, RADA16-1, and RAD/RMOTS-c. (B–C) Growth curves of NP-MSCs were generated under various subgroup conditions in both normal medium and in the TBHP150μM environment. (D–F) The expression of functionally related genes and proteins was analyzed in treatment groups using RAD/RMOTS-c hydrogel, RADA16-I, and MOTS-c.(∗, P < 0.05,∗∗,P < 0.01,∗∗∗,P < 0.005,∗∗∗∗,P < 0.001,ns, no significant).

NP-MSCs were inoculated into RADA16-I, RAD/RMOTS-c, and MOTS-c-containing media, followed by a 48-h incubation period for each group. Cytotoxicity testing of the two scaffold materials and the MOTS-c containing medium revealed a small number of dead cells stained red with PI, observed within the peptide hydrogel scaffold materials and MOTS-c medium in both groups (Fig. 10A). The results indicated that the self-assembled peptide nanofiber scaffolds RADA16-I and RAD/RMOTS-c materials, and MOTS-c exhibited no significant cytotoxicity.

### 3.16 Effects of different TBHP concentration and materials on the growth of NP-MSCs

Fig. 10B shows that in the TBHP 0 μM culture environment, the viability and growth of NP-MSCs in the MOTS-c and RAD/RMOTS-c groups were significantly improved over time compared to the RADA16-I group (p < 0.001). Additionally, both MOTS-c and RAD/RMOTS-c significantly increased the proliferation rate of NP-MSCs in the TBHP 150 μM culture environment, indicating the promotion of NP-MSCs regeneration by both RAD/RMOTS-c and MOTS-c.

### 3.17 RAD/RMOTS-c regulates the expression of ECM-related genes and proteins

NP-MSCs were cultured in RAD/RMOTS-c hydrogel along with MOTS-c-containing medium for 48 h, with RADA16-I serving as the control. As shown in Fig. 10D–F, the results demonstrated that NP-MSCs secreted significantly higher gene and protein levels of collagen II and proteoglycans and significantly lower gene and protein levels of collagen I in the RAD/RMOTS-c treatment group compared to both RADA16-I hydrogel scaffold material and MOTS-c groups, with statistically significant differences (p < 0.005). This suggests that the RAD/RMOTS-c hydrogel scaffold material effectively enhances type II collagen and proteoglycan secretion while reducing type I collagen expression by NP-MSCs, thus favoring cellular homeostasis and functional expression.

### 3.18 Fiber ring puncture needle preparation and puncture localization in rats

**Fig. 11:**
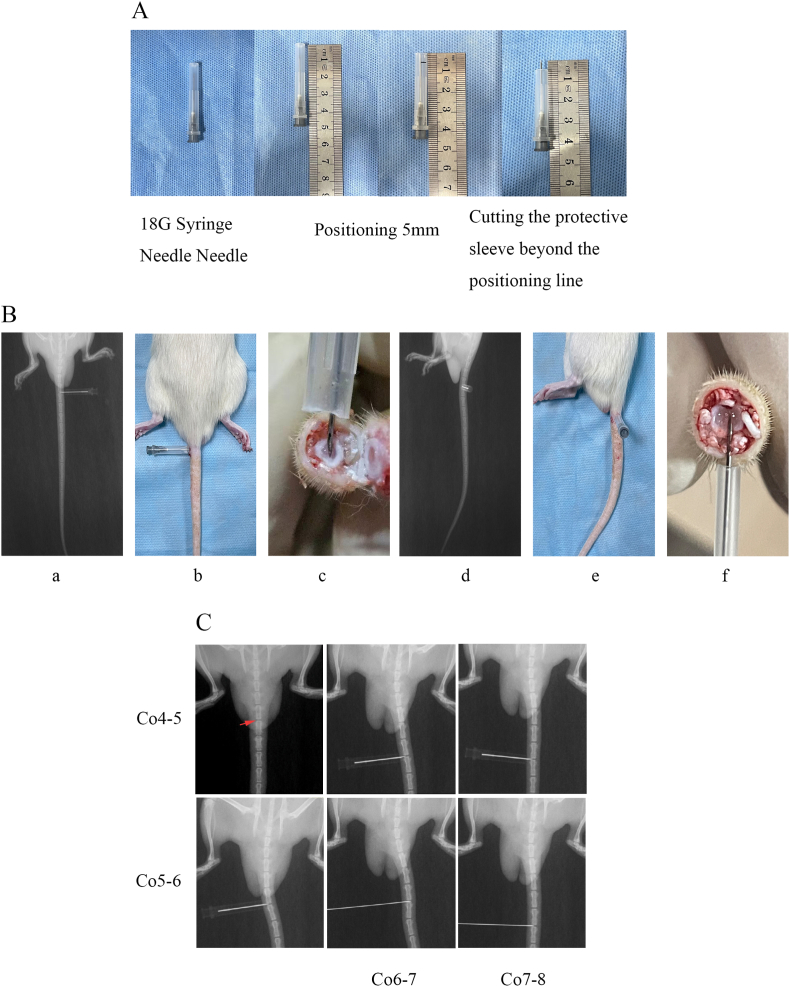
(A) Preparation of the puncture needle for rat disc animal modeling. (B) X-ray positioning model for posterior and lateral punctures and microsyringe administration in rats. Figure B(a) depicts an orthographic view of the rat. Figure B(b) shows the actual image of the rat in an upright position. Figure B(c) displays the actual image of a 5 mm needle insertion. Figure B(d) presents a lateral X-ray film. Figure B(e) exhibits a lateral physical image. Figure B(f) illustrates the actual process of microsyringe administration. (C) Modeling puncture of four intervertebral discs between Co4-8 vertebrae in SD rats and X-ray localization of administration.

X-ray frontal and lateral views, as well as physical images of the rat intervertebral discs, were obtained following puncture and modeling using an 18G syringe needle, as depicted in Fig. 11B (a, b, d, e). This method ensured accurate puncturing to the central position of the intervertebral disc with a depth limit of 5 mm using an 18G needle, which was then slowly withdrawn post-disc damage. Fig. 11B (c) illustrates the physical placement of the 18G needle within the central NP of the annulus fibrosus, passing through the skin and surrounding ligaments. Fig. 11B (f) demonstrates the injection of 5 μL of RADA16-I or RAD/RMOTS-c drug using a 5 mm microinjector with depth limitation, reaching the central position of the NP through the skin and surrounding tissues. X-ray localization results for the normal, IDD, RAD/RMOTS-c, and RADA16-I groups were assigned to Co4-5, Co5-6, Co6-7, and Co7-8, respectively, corresponding to the four segments of the intervertebral discs, and treatments were administered accordingly, as shown in Fig. 11C.

### 3.19 Magnetic resonance imaging (MRI) analysis

**Fig. 12:**
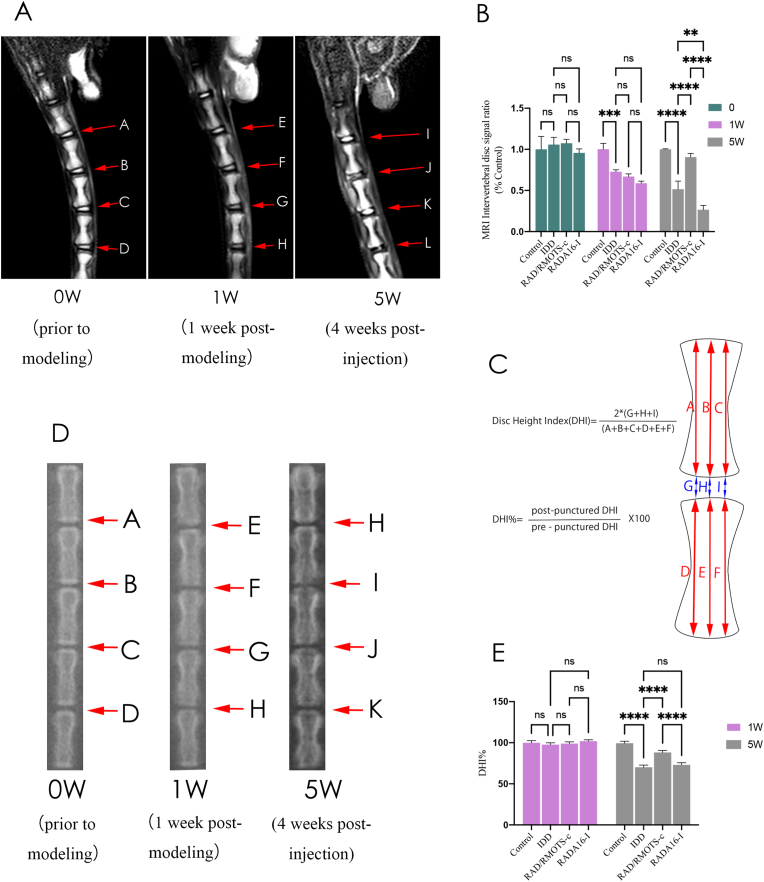
(A–B) MRI scanning results and data analysis of rat coccyx. (C) Diagram of calculated model of intervertebral disc height index and DHI%. (D–E) Rat coccyx X-ray scanning results and data analysis. (∗,P < 0.05,∗∗,P < 0.01,∗∗∗,P < 0.005,∗∗∗∗,P < 0.001,ns, no significant.

MRI was utilized to evaluate T2-weighted signal intensity and histological changes in the NP. Fig. 12A and B demonstrates no significant difference in disc signals of the control group at 0, 1, and 5 weeks. At 1 week post-modeling, the MRI indices in IDD, RAD/RMOTS-c, and RADA16-I groups were significantly lower than those in the control group. At 4 weeks post-injection, the MRI indices in the IDD group were significantly lower than those in the control group (p < 0.001), indicating successful modeling of annulus fibrous puncture injury and significant degenerative changes in the disc. The MRI indices in the RAD/RMOTS-c group were significantly higher than those in the IDD and RADA16-C groups (P < 0.01), indicating a notable difference in outcomes. This suggests that RAD/RMOTS-c treatment significantly improves degenerative disc disease caused by puncture injury to the annulus fibrosus of the intervertebral disc. Additionally, the MRI index of the RADA16-I group was significantly lower than that of the IDD group (p < 0.001), possibly due to increased pressure on disc tissues in the RADA16-I treatment group. The cellular therapeutic efficacy of RADA16-I was not evident, resulting in stress injuries to NP cells and exacerbating apoptosis of both NPCs and NP-MSCs.

### 3.20 Intervertebral disc height measurement

Measurements of the disc height index (DHI) were taken prior to modeling (0W), 1 week after modeling (1W), and 4 weeks after injection (5W) using radiography. Following the method described by Masuda et al. [31], as shown in Fig. 12C, DHI was determined by dividing the height of the intervertebral disc (IVD) by the height of the adjacent vertebrae. DHI% is calculated by dividing post-puncture DHI by pre-puncture DHI and then multiplying by 100 %. As depicted in Fig. 12D and E, the results indicated no significant difference in DHI% between groups at 1 week after modeling, with DHI% remaining stable across groups. At 4 weeks post-injection, DHI% was significantly lower in the IDD group compared to the control group, with a statistically significant difference. Additionally, no statistically significant difference was observed between the IDD group and the RADA16-I group at 4 weeks post-injection. However, the RAD/RMOTS-c group exhibited a significantly greater reduction in DHI% compared to the IDD group (P < 0.05). This suggests that RAD/RMOTS-c treatment can significantly alleviate the reduction in disc height caused by degenerative disc disease.

### 3.21 Histological analysis

**Fig. 13:**
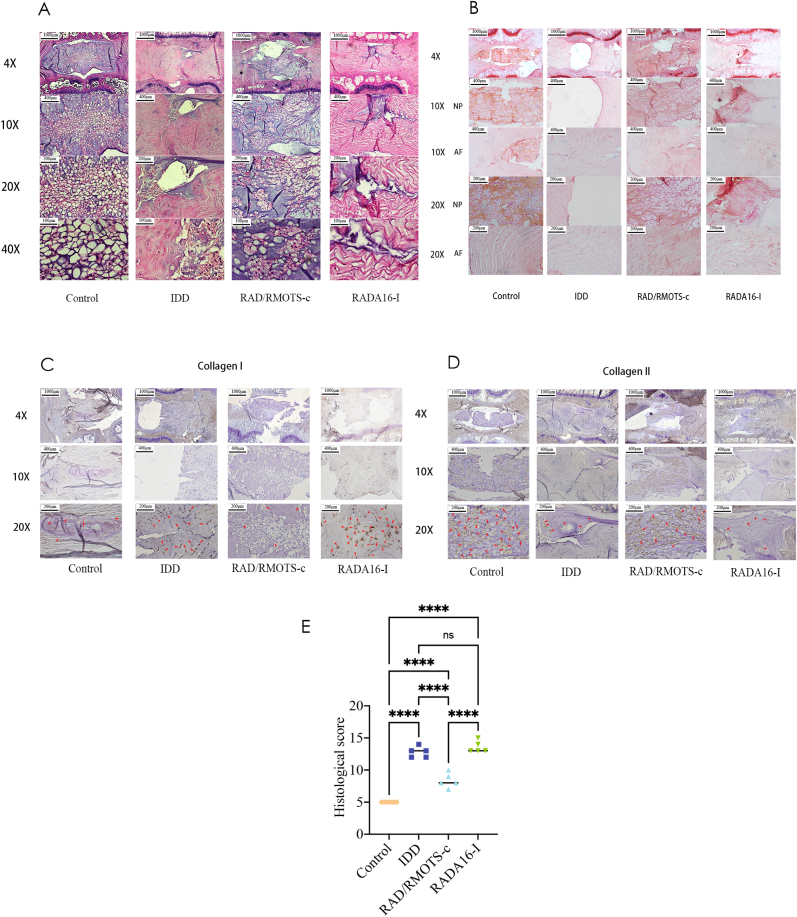
(A-D)Representative HE staining,SO staining and immunohistochemical staining of Collagen I and Collagen II expression in each group. Magnification: 4× (scar bar = 500 μm),10 × (scar bar = 200 μm), 20 × (scar bar = 100 μm) and 40 × (scar bar = 50 μm). The red arrows indicate corresponding positive cells. (E) Histological grades were assessed according to the HE staining (n = 5 for each group). Data were presented as the mean ± SD. (∗∗∗∗, P < 0.001; ns, no significant.). (For interpretation of the references to colour in this figure legend, the reader is referred to the Web version of this article.)

Histopathologic sections stained with hematoxylin and eosin (H&E) and SO can illustrate the structure of the intervertebral disc, comprising the NP, annulus fibrosus, cartilaginous endplates, and adjacent vertebrae. As shown in Fig. 13A, HE staining revealed well-organized and homogeneous NP tissue in the control group. Conversely, NP tissue in the IDD and RADA16-I groups exhibited destruction or even complete disappearance, along with the loss of the gel-like properties characteristic of the NP. Additionally, AF tissue showed disorganization and disruption of its concentric lamellar structure with fibrous breaks. In the IDD and RADA16-I groups, the NP tissue nearly vanished and was replaced by fibrillar connective tissues 4 weeks post-injection. The distribution of NP and ECM structures was distinctly visible in the RAD/RMOTS-c group, exhibiting superior quantity and organization compared to the IDD and RADA16-I groups.

As shown in Fig. 13B, SO staining indicated a significant decrease in proteoglycan levels in the IDD and RADA16-I groups, whereas the control and RAD/RMOTS-c groups displayed stronger staining compared to the IDD group. At 4 weeks post-injection, the histological scores of all groups were notably higher than those of the control group, whereas the RAD/RMOTS-c group exhibited a lower score compared to the RADA16-I and IDD groups (Fig. 13E, p < 0.001). Except for the RAD/RMOTS-c and control groups, the histological scores of the IDD group were equivalent to those of the RADA16-I groups.

### 3.22 Immunohistochemistry-based verification of the low expression of collagen I and high expression of collagen II in RAD/RMOTS-c treatment group

Immunohistochemical staining for collagen II and collagen I in the annulus fibrosus and NP tissues was carried out in order to reveal the state of the ECM and assessing the extent of degenerative disc disease.

As shown in Fig. 13C and D, the expression of collagen II and collagen I were detected at 4 weeks after injection. The expression of collagen I, stained dark brown, was distributed in the IDD and RADA16-I groups. However, there were fewer areas of positive staining in the control and RAD/RMOTS-c groups. This indicates that RAD/RMOTS-c can significantly reduce collagen I expression in the extracellular matrix.

Collagen II is an important component of the NP and annulus fibrosus that provides mechanical strength. Decrease in collagen II may lead to degeneration of the extracellular matrix and intervertebral disc. As shown in Fig. 13D, collagen II was stained dark brown and was distributed in the control and RAD/RMOTS-c groups. Since the normal structure of NP disappeared in the IDD and RADA16-I group, only the smallest area of NP tissue was found to be positive for collagen II. These results suggest that that the ECM had degenerated and remodeled. It also suggests that annulus fibrosus puncture can degrade the extracellular matrix and mimic disc degeneration.

### 3.23 RAD/RMOTS-c increased the expression level of collagen II and aggrecan and decreased the expression level of collagen I

**Fig. 14:**
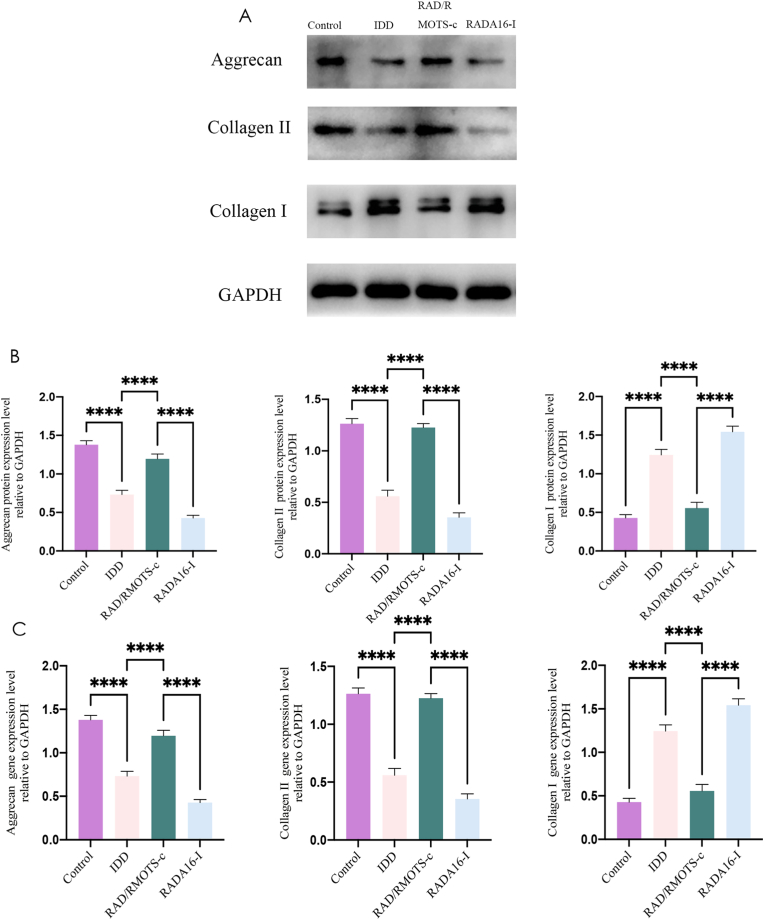
(A–B) Western blot analysis and the quantitative statistical analysis showed the protein levels of Aggrecan, Collagen I and Collagen II expression. GAPDH was used as an internal control. (C) The gene expression levels of Aggrecan, Collagen I and Collagen II were measured by RT-PCR analysis. GAPDH was used as an internal control. Data were presented as the mean ± SD.(∗,P < 0.05,∗∗,P < 0.01,∗∗∗,P < 0.0005,∗∗∗∗,P < 0.0001,ns, no significant).

As shown in Fig. 14, the expression levels of collagen II and aggrecan genes and proteins was found to be lower in the IDD and RADA16-I group than the control group, but the collagen I gene and protein showed a higher expression in the control group. The RAD/RMOTS-c treated group showed a significantly higher expression of ECM-related genes and proteins, such as collagen II and aggrecan, than the IDD group. The expression of collagen I genes and proteins in the RAD/RMOTS-c treated group was lower than in the IDD group; this result was statistically significant. The expression of collagen II and aggrecan genes and proteins in the RADA16-I-treated group was significantly lower than in the IDD group, and the expression of collagen I was higher than in the IDD group. These results indicate that the degeneration of the intervertebral disc in the RADA16-I-treated group was more serious.

## 4 Discussion

### 4.1 Rationale for the selection of and characteristics of NP-MSCs investigated in this study

Presently, MSCs employed in the treatment of IDD predominantly encompass bone marrow-derived MSCs (BMSCs) [[32], [33], [34]], adipose-derived MSCs [35,36], synovial-derived MSCs [37,38], muscle-derived MSCs [39], and embryonic-derived MSCs [40]. Furthermore, the isolation and extraction of exogenous MSCs from the umbilical cord have been used for the repair and regeneration of disc tissue. Following direct transplantation of these stem cells into the injured disc, in situ co-culture of stem cells and disc cells facilitates stem cell redifferentiation and promotes regeneration of the NP tissue. However, in the study, a formidable challenge was identified. As disc tissue degenerates, the microenvironment within the extracellular matrix of the disc becomes characterized by hypoxia, hypoglycemia, acidity, high osmotic pressure, elevated mechanical load pressure, and increased inflammatory factors. These alterations in the microenvironment exacerbate disc degeneration and inhibit the survival and differentiation of stem cells, ultimately resulting in transplanted stem cells being subject to senescence or apoptosis. Further exploration of stem cells capable of withstanding the harsh microenvironment revealed the existence of stem or progenitor cells residing in both the NP and the annulus fibrosus or cartilage endplates [6]. Endogenous NP-MSCs originating from medullary tissues exhibited superior resilience to the cellular microenvironment. They were capable of handling high osmotic pressure, hypoxia, and acidic conditions. Thus, it is widely acknowledged that NP-MSCs stand out as the most promising candidates for stem cell-based therapies targeting IDD. Subsequent biotherapeutic endeavors may concentrate on fostering the development of in situ NP-MSCs. However, inflammation, oxidative stress, and annulus fibrosus rupture may cause the production of ROS, and the accumulation of ROS in the disc will lead to the senescence and apoptosis of NP-MSCs, and eventually to irreversible IDD. Therefore, inhibition of oxidative stress-induced senescence and apoptosis of NP-MSCs may be of great significance in alleviating IDD. In the present study, TBHP was used to trigger oxidative stress in NP-MSCs, which is widely accepted as an apoptosis and senescence in vitro model.

### 4.2 Effect of MOTS-c on the activity of NPMSCs

MOTS-c, encoded by functional short open reading frames in the mitochondrial DNA (mtDNA), discovered by Lee et al. [18] in 2015, promotes the biosynthesis of the endogenous AMP analogue AICAR, which activates AMPK, and may have potential for preventing type 2 diabetes by slowing the aging process [41]. In addition, MOTS-c possesses a wide range of protective effects, such as anti-inflammatory, antioxidant, anti-senescence and anti-apoptotic effects [25,[42], [43], [44], [45]]. The idea that MOTS-c may be associated with longevity is of particular interest [46]. Research on the effects of MOTS-c on NP cells or NP-MSCs in IDD has not been proposed or studied. Therefore, in this pioneering study, we focused on the role of MOTS-c in relieving oxidative stress in NP-MSCs and aimed to elucidate its underlying mechanisms.

The results of our present study revealed that pretreatment with MOTS-c can significantly decrease senescence, ROS levels, and apoptosis induced by oxidative stress in TBHP, and the inhibition of proliferation was also significantly alleviated but still did not improve to the level of normal NP-MSCs. These results indicate that MOTS-c can counteract oxidative stress, thereby protecting NP-MSCs from oxidative stress damage.

Senescence is known to accelerate the decline in viability and function of intervertebral disc NP cells while also increasing oxidative stress within these cells. Cell senescence exhibits the characteristics of decreased cell viability and the loss of proliferation capacity. The main molecular pathways involved in senescence are p53-p21 CIP 1, p16 INK 4a, NF-κB, and MAPK, and the mechanisms involved in IDD include mechanical, oxidative, genotoxic, and inflammatory stress, as well as nutrient deprivation [[47], [48], [49], [50]]. During the process of IDD, age-related damage and degenerative external stimuli activate the p53 p21 Rb and p16 Rb pathways, inducing intervertebral disc cell aging [51]. Induction and maintenance of aging by p16 Ink 4a and the conditional loss of p16 Ink 4a reduce cell apoptosis, limit SASP phenotype, and alter matrix homeostasis of intervertebral disc cells [52]. p53 plays a role as a transcription factor in cell-autonomous responses, such as cell cycle control, DNA repair, apoptosis, and cellular senescence. High levels of p53 are known to stimulate a range of p53-responsive genes, leading to cell cycle arrest or apoptosis [53,54]. The p16-mediated senescence acts through the retinoblastoma (Rb) pathway, inhibiting the action of the cyclin dependent kinases and leading to G1 cell cycle arrest [55].

Oxidative stress leads to high production of ROS within NP cells while also reducing antioxidant levels [56]. The primary function of the IVD is stress distribution. However, excessive compression induces oxidative stress, mitochondrial dysfunction, and apoptosis of NP cells. Consequently, many scholars regard oxidative stress as the primary causative factor in the intricate pathophysiological processes and pathogenic mechanisms of IDD [57,58]. Maintaining a balance between ROS and antioxidants is crucial for normal function and survival of IVD cells. Excessive ROS levels can damage cellular macromolecules, such as nucleic acids, lipids, and proteins, impacting normal cellular activities and functions. Additionally, excessive ROS can hasten IDD progression by inducing inflammatory, apoptotic, and senescence pathways. Based on this rationale, in this study, we simulated an in vitro model of IDD by inducing oxidative stress in NP-MSCs using TBHP [59,60]. We observed a significant decrease in the regenerative capacity of NP-MSCs under TBHP-induced oxidative stress, along with increased ROS generation and rates of cellular senescence and apoptosis. Additionally, functionally related proteins (collagen II and aggrecan) in NP-MSCs were significantly reduced, confirming that oxidative stress severely damages the structure and function of NP-MSCs.

Other antioxidant drugs similar to MOTS-c, such as andrographide, sinomenine, marein, baicalin, and quinazoline, can inhibit IDD by suppressing ROS accumulation in the NPCs [[61], [62], [63], [64], [65]]. Curcumin, 6-GIN, Evodiamine and Tetradrine induce autophagy and enhance autophagy flux. By enhancing autophagy to inhibit excessive ROS production and mitochondrial dysfunction, TBHP-induced apoptosis, aging, and ECM degradation in NP cells were improved [57,[66], [67], [68]]. The effectiveness of ozone injection into intervertebral discs in IDD rats (O_3_ through PI 3 K/Akt/NF- κ signaling pathway) inhibits the progression of IDD [69]. The regulation of oxidative stress damage is crucial for the treatment of IDD.

Bcl-2 (anti-apoptotic protein) and Bax (pro-apoptotic protein) are the two proteins used to evaluate cell susceptibility to apoptosis. The activity of the caspase pathway is promoted by Bax but inhibited by Bcl-2 and inhibits cell apoptosis. In the present study, we found that MOTS-c significantly inhibited the TBHP-induced up-regulation of p53, p16, Caspase-3, and Bax expression levels and restored the down-regulation of Bcl-2 expression levels.

Therefore, these results suggest that MOTS-c can protect NP-MSCs from TBHP-induced injury.

Previous research has identified significant variations in the effective concentration of MOTS-c across various cell types. Specifically, MOTS-c has been observed to stimulate cell proliferation in osteoblasts and neuronal cells within the range of 0–2 μM while exhibiting an inhibitory effect on neuronal cell activity at 4 μM [70,71]. Investigations into stem cell behavior revealed that MOTS-c did not influence bone marrow MSC activity at concentrations ranging from 0 to 1 μM but promoted bone marrow MSC proliferation within the range of 1–4 μM [72]. Similarly, the proliferation of placental MSCs was stimulated by MOTS-c within the range of 1–10 μM [22]. Furthermore, MOTS-c demonstrated significant antioxidant and anti-inflammatory effects on cardiomyocytes when applied within the range of 0–50 μM [73]. The effective concentration for protective effects of MOTS-c varied considerably among different cell types, with stem cells showing a significantly broader range of protective concentrations compared to adult cells. This study investigated the impact of MOTS-c concentrations ranging from 0 to 250 μM on the activity of NP-MSCs. Results indicated that MOTS-c exhibited no significant cytotoxicity on NP-MSCs at concentrations up to 100 μM. Additionally, NP-MSCs maintained maximum cellular activity following oxidative stress injury when treated with 150 μM TBHP after pretreatment with 100 μM MOTS-c. Consequently, the subsequent MOTS-c experiments in this study were conducted at a concentration of 100 μM.

### 4.3 Possible mechanism analysis of the effect of MOTS-c on NPMSCs activity

Several studies on MOTS-c have found that it can be expressed through the AMPK, Nrf2/ARE, and NF-κB signaling pathways, all of which play a role in reducing cellular oxidative damage and protecting the antioxidant system [71,73,74]. MOTS-c's nuclear translocation capability is also influenced by AMPK. During stressful conditions, MOTS-c translocates to the nucleus to directly modulate the expression of adaptive nuclear genes and enhance cellular homeostasis [43].

MOTS-c significantly improves mitochondrial homeostasis by decreasing oxygen consumption and ROS production. High glucose induces excessive ROS and promotes mitochondrial damage, leading to dose-and time-dependent apoptosis of rat CEP cells. Appropriate glucose control may be the key to prevent IDD in patients with diabetes [75]. The initial application of MOTS-c was mainly to alleviate hyperglycemia and insulin resistance [76,77], regulate PGC-1 expression through the AMPK signaling pathway, reduce insulin resistance, and enhance glucose metabolism [78].

The study also indicates a close interaction between SIRT1 and AMPK in the regulation of energy, metabolism, aging, and other functions. They mutually enhance each other's activities. Pharmacological activation of AMPK enhances mitochondrial biogenesis and function in OA chondrocytes while also triggering the SIRT1-PGC1α signaling pathway, which helps in slowing down the aging process of these chondrocytes. Moreover, AMPK activation delays cell senescence by promoting NAD+/SIRT1 and boosting autophagy. Previous research [79] has shown that SIRT1 overexpression can impede NP cell senescence, stimulate cell proliferation, and prevent cell apoptosis in IDD. Hence, investigating whether MOTS-c elicits similar effects on NP-MSCs was the central focus and objective of this study. We postulated that MOTS-c alleviates oxidative stress-induced injury in NP-MSCs through the activation of the AMPK-SIRT1 signaling pathway.

MOTS-c significantly activates AMP-activated protein kinase, the main target pathway of MOTS-c, and inhibits its antagonistic effector, mTORC1. MOTS-c significantly enhances mitochondrial homeostasis by reducing oxygen consumption and ROS production [22]. Shen C et al. found that MOTS-c could protect against H_2_O_2_-induced inflammation and oxidative stress in H9c2 cells by inhibiting NF-κB and activating the Nrf2/ARE pathways [73]. Wen et al. also found that MOTS-c prevented apoptosis in pulmonary microvascular endothelial cells by inhibiting the expression of inflammatory and oxidative stress factors through the Nrf2 and MAPK pathways [80]. Under normal conditions, the pro-longevity enzyme, 5′ AMP-activated protein kinase (AMPK), is dedicated to the homeostasis of metabolism and autophagy for removal of damaged cellular compartments and molecules [81].

The AMPK signaling pathway plays a regulatory role in autophagy and aging through its interactions with multiple key molecules, including mTOR, ULK1, FOXO, p53, SIRT1, and NF-κB [82]. The interaction of AMPK with SIRT1 is particularly crucial in this process. Cells produce high levels of ROS during adipogenic differentiation. Inhibiting SIRT1 during differentiation leads to impaired oxidative stress response. In mice with MSC-specific knockout of the SIRT1 gene, aging-related markers, such as p16 and β-galactosidase activity, are elevated in inguinal adipose tissue [83]. SIRT1, an NAD (+)-dependent Class III histone deacetylase, is a multifunctional protein that acts on various substrates—including p53, forkhead box (FOXO) transcription factors, PGC-1α, NF-κB, and histones—mediating processes, such as stress response, cellular metabolism, and aging [84,85]. SIRT1 was initially considered a potential tumor promoter as it negatively regulates tumor suppressor p53 and other tumor suppressor factors [86]. Cellular senescence, defined as the permanent withdrawal of cells from the cell cycle in response to stress, is primarily governed by tumor suppressor proteins, such as p53 and retinoblastoma protein (RB), forming a critical anti-cancer mechanism. Notably, p53 can be deacetylated by a protein complex containing histone deacetylase 1 (HDAC1) [87].

The AMPK/SIRT1 pathway plays a crucial role in regulating various biological processes, including cell proliferation, differentiation, pyroptosis, apoptosis, autophagy, inflammation, oxidation, senescence, and adipose remodeling, by interacting with upstream and downstream proteins under both physiological and pathological conditions [[88], [89], [90], [91], [92], [93], [94]]. AMPK regulates energy expenditure by modulating NAD + metabolism and SIRT1 activity [95,96]. This has been shown in a variety of contexts. Berberine modulates deacetylation of PPARγ to promote adipose tissue remodeling and thermogenesis via AMPK/SIRT1 pathway [89]. Quercetin activates the AMPK/SIRT1 axis to improve amyotrophic lateral sclerosis and contributes to apoptosis in A549 and H1299 lung cancer cells [90,97]. Pterostilbene suppresses oxidative stress and allergic airway inflammation through AMPK/SIRT1 and Nrf2/HO-1 pathways [98]. The activation of AMPK/SIRT1 signaling pathway has been found to improve mitochondrial energy metabolism and oxidative stress [[99], [100], [101]]. Mounting evidence that both SIRT1 and AMPK play important roles in cell fate determination of MSCs exists [94]. Activation of AMPK/SIRT1 pathway could alleviate neurodegeneration in aging [[102], [103], [104]]. Metformin mitigates cholesterol accumulation via the AMPK/SIRT1 pathway to protect osteoarthritis chondrocytes and regulate plasma lipids [[105], [106], [107]]. ROS and AMPK-SIRT1 perform a multi-regulatory network [108]. Orientin downregulates oxidative stress-mediated endoplasmic reticulum stress and mitochondrial dysfunction through AMPK/SIRT1 pathway in rat NP cells in vitro and attenuates IDD in vivo [109]. MOTS-c regulates various biological processes, including inflammatory responses, oxidative stress, and aging, via the AMPK/SIRT1 signaling pathway [22,110,111]. In this study, we discovered that MOTS-c mitigated TBHP-induced inhibition of the AMPK/SIRT1 signaling pathway, consequently decreasing ROS production and enhancing the activity and function of NP-MSCs. Nevertheless, when the SIRT1 pathway was blocked using a SIRT1 inhibitor, the protective impact of MOTS-c on NP-MSCs was diminished or abolished. Consequently, our findings imply that MOTS-c may mitigate TBHP-triggered senescence and apoptosis in NP-MSCs while safeguarding their functionality through activation of the AMPK/SIRT1 signaling pathway.

### 4.4 Gelling properties and biocompatibility analysis of RADA16-I conjugated by MOTS-c

MOTS-c, like other peptide drugs, can undergo rapid degradation and become ineffective due to the action of various enzymes in bodily fluids, or it may induce cytotoxic effects as a result of excessive local concentrations [112,113]. A method of delivering a sustained dose of MOTS-c to the disc tissue is urgently needed to facilitate the IVD repair process.

RADA16-I is a typical self-assembling peptide that has been clinically used in surgical hemostatic agents, wound dressings, and tissue engineering scaffolds [114,115]. It can form 3D hydrogel scaffolds in neutral pH and saline solutions containing more than 99 % water, with excellent biocompatibility and low immunogenicity. Its degradation products are amino acids, which reduce the likelihood of inflammatory reactions and have little effect on the normal healing process of damaged tissue. Self-assembling peptides can be reduced to short peptide sequences and subsequently degraded to individual amino acids, constituting a group of naturally biodegradable materials [116]. Specific functional peptides can modify either the C-terminus or N-terminus of RADA16-I, preserving its original function while enhancing the capabilities of RADA16-I self-assembled hydrogels [117]. Studies on sustained release of RADA16-I bound peptides have shown that the release of its linked peptides can be sustained up to 2 days or even longer [[118], [119], [120]]. Consequently, we engineered a hydrogel scaffold to serve as a delivery system for MOTS-c, aiming to optimize therapeutic approaches for intervertebral disc regeneration. In our investigation, we utilized RAD/RMOTS-c functionalized self-assembled peptide hydrogel both as a carrier for MOTS-c and scaffold matrix for NP-MSCs cells, targeting disc repair. The gelation process can enhance the self-assembling process and form interwoven structures resembling the ECM microenvironment [121].

At RADA16-I peptide concentrations in the range of 3–6.5 mg/mL, fibrous aggregates form a weak gel network, which can be disrupted on dilution [122]. Thus, in our experimental setup, the initial concentration of RADA16-I was 10 mg/mL. As RMOTS-c peptide lacked the ability to self-assemble into a gel structure, it was co-assembled with RADA16-I in a 1:1 ratio. This ensured a minimum concentration of 5 mg/mL of RADA16-I, maintaining the gel-forming capability of RAD/RMOTS-c. Higher-concentration hydrogels provide better mechanical properties, but lower-concentration hydrogels are more conducive to stem cell differentiation, and the compression modulus of 5 % GelMA/PRP is close to that of normal NP tissue [123].

In agreement with the findings by Sun et al. [124], the RADA16-I and RAD/RMOTS-c viscoelasticity examinations in our experiments also featured hydrogels with significantly higher G″ than G′. Similar to the results of Sun et al. [125] who found that the storage modulus of R3 (n-RADARADARADA-GGAGGS-c) was 0.5 times higher than that of RADA16-I, we found that the viscosity of the two gels, RADA16-I and RAD/RMOTS-c, is about 217 Pa-s and 131 Pa-s, respectively, and the storage modulus of RAD/RMOTS-c is about 0.6 times that of RADA16-I. The gel-forming stability of the gel system after splicing MOTS-c was reduced compared with that of RADA16-I. The modulus decreased with increasing concentrations of the RMOTS-c peptide, but it was still able to maintain the 3D structure after gel formation. The storage modulus of NP tissue is about 64 ± 28 kPa, and the loss modulus is about 24 ± 11 Kpa [126]. Our study found similar energy storage modulus and loss modulus for RAD/RMOTS-c.

Hydrogels for disc therapy should be elastic, permeable, and swellable [[127], [128], [129]]. In order to maintain intradiscal pressure (limited space inside the IVD), the swelling rate of the hydrogel should be controlled. The swelling rate of the hydrogel in our study was significantly low, which is reassuring. RAD/RMOTS-c was found to have low swelling properties at 0 % and 10 % strain. All hydrogels presented a porous and homogeneous structure with interconnected pore units of about 100 nm in diameter. RAD/RMOTS-c hydrogel has a homogeneous structure and interconnected porous structure with uniform pore distribution and high porosity, which not only provides enough space for cell migration and proliferation but also facilitates cell growth and exchange of nutrients and cellular metabolites in the hydrogel.

RADA16-I when exposed to pH 1.0 and 4.0 was still able to assume a typical β-folded structure and self-assemble into long chains. Similar to the effect of pH, RADA16-I was still able to present a typical β-folded structure with 46 % content when thermal denaturation occurred at 25–70 °C [45]. RADA 16-I protofibres were stable at pH 2.0–4.5 with lengths of 200–400 nm and diameters of 10 nm [130]. However, when exposed to pH 13.0, RADA16-I dramatically loses its β-folded structure and assembles into different small-sized globular aggregates. The microenvironment that often occurs in intervertebral disc tissues during IDD is acidic. In this acidic environment, RADA16-I can still maintain a relatively stable β-folded structure and function.

RADA16-I can spontaneously form a hydrogel in NaCl solution and self-assemble into scaffolds with β-sheet structures. By neutralizing the pH and increasing the ion concentration in the serum, it is possible to create new structures with a higher modulus of elasticity. Consequently, RADA16-I and RAD/RMOTS-c could form more stable hydrogel-like structures in serum-containing medium or in vivo animal experiments. When RADA16-I comes into direct contact with blood, instead of a secondary β-sheet structure, a tertiary structure is formed, which in turn enhances the elasticity of the hydrogel in the presence of blood [131].

Compatibility analysis showed that the composite hydrogel could support the long-term survival and proliferation of ADSCs, and the cells were evenly distributed and well-structured in the composite hydrogel. In conclusion, RAD/RMOTS-c composite hydrogel is an ideal carrier of MOTS-c for the treatment of senescence and apoptosis of NP-MSCs in IDD.

### 4.5 Analysis of in vivo validation results in the rat model

In vivo animal experiments are important preclinical tools for biomedical research. Among the animal models of oxidative stress-related IDD, the intervertebral disc puncture model is currently considered the model of choice [113,132,133]. In our study, we also used needle puncture to construct an animal model of oxidative stress-related IDD. This is mainly due to the fact that there is still a lack of animal models of IDD after specific oxidative stress challenge. We indirectly evaluated the antioxidant, anti-senescence, and anti-apoptotic effects of the RAD/RMOTS-c hydrogel in vivo based on its therapeutic outcomes.

Upon reviewing the MRI and X-rays of the rats 1 week after IDD puncture modeling, we observed a mild degree of disc degeneration. This suggests that IDD modeling was successful after just 1 week, obviating the need to wait for 2 weeks or more. There are two primary reasons for this. First, waiting for 2 weeks or more after puncture may result in irreversible damage to the disc tissue, thereby rendering the injection of tissue-engineered hydrogel ineffective. Second, disc degeneration is not notably severe in most clinical patients. Therefore, we only need to simulate mild disc degeneration after modeling in order to recapitulate conditions realistically similar to those observed in human patients with IDD. Early detection and treatment can significantly improve prognosis. The residual NP cells in the disc tissue can induce NP-MSC differentiation and enhance the efficacy of MOTS-c. Once severe disc degeneration occurs, it is often irreversible and necessitates surgery for symptomatic relief.

Our study is different from other studies using other tissue engineering repair techniques. Because the combined use of stem cells and functional hydrogel peptides for disc repair is complex, and implanted stem cells within the intervertebral disc tend to undergo rapid apoptosis, our experiment instead focused on enhancing the reparative capacity of resident NP-MSCs. By using a functional hydrogel peptide, we aimed to improve the oxidative stress resistance of NP-MSCs and promote in situ repair of the intervertebral disc microenvironment.

Proper function of the intervertebral disc depends on healthy ECM structure and composition. When IDD occurs, chronic metabolic disorders occur in the extracellular microenvironment within the intervertebral disc. Sustained oxidative stimulation by annulus fibrosus puncture or TBHP can cause the balance between oxidation and antioxidants in the intervertebral disc to be broken, and ROS and sustained inflammation cause the degradation of the extracellular matrix. In our in vitro cell experiments and in vivo animal experiment results, proteoglycans and collagen II were gradually replaced by collagen I in NP-MSCs and intervertebral disc tissue after TBHP and puncture injury. The IDD group showed severe degenerative lesions and structural disorder of the intervertebral disc, decreased intervertebral disc height, decreased intervertebral disc water content, reduced or disappeared NP matrix content, and rupture of the annulus fibrosus. In the RAD/RMOTS-c treatment group, the degenerated intervertebral disc was significantly repaired, the intervertebral disc height and water content were significantly increased, and the structure and function of the NP and annulus fibrosus were relatively complete. The lesions in the pure RADA16-I group were similar to those in the IDD-treated group, with severe IDD occurring. The aggrecan and type II collagen were gradually replaced by type I collagen.

### 4.6 Limitations of the study

Although this study provides valuable insights into IDD therapeutics, some limitations warrant consideration. First, the mechanistic validation of AMPK/SIRT1 signaling in NP-MSCs relied on pharmacological inhibition rather than genetic approaches (e.g., siRNA-mediated gene silencing or CRISPR-based knockout), which could more definitively establish causal relationships within the pathway. Second, the rat IDD model induced using needle puncture primarily mimics acute mechanical injury but fails to recapitulate the chronic, multifactorial pathogenesis of human IDD involving age-related metabolic dysfunction, sustained inflammatory cascades, and cumulative mechanical loading. Third, although the RAD/RMOTS-c hydrogel demonstrated basic biocompatibility, critical pharmacokinetic parameters—including MOTS-c release kinetics under physiological shear stress, peptide degradation profiles, and structure-function relationships under dynamic loading—remain to be quantitatively characterized. Fourth, the in vivo evaluation focused on structural and radiological outcomes (DHI%, MRI signals) without assessing molecular-level therapeutic effects, such as oxidative stress markers (e.g., 8-OHdG, SOD activity), pro-inflammatory cytokine levels (IL-1β, TNF-α), or mechanotransduction pathways (YAP/TAZ) in treated discs. Finally, the absence of multi-omics validation (transcriptomic/proteomic analyses) limits the depth of mechanistic understanding regarding MOTS-c's systemic effects on disc microenvironment remodeling. Addressing these gaps through advanced genetic models, humanized disease simulations, and longitudinal molecular profiling will strengthen the clinical relevance of this therapeutic strategy.

## 5 Conclusions

In conclusion, this study establishes a dual-functional strategy for intervertebral disc regeneration by integrating mitochondrial protection with structural reinforcement through a rationally designed bioactive hydrogel system. The RAD/RMOTS-c self-assembling peptide hydrogel not only serves as a mechanically adaptive scaffold mimicking the native disc microenvironment but also achieves sustained MOTS-c delivery to counteract oxidative stress-driven NP-MSC degeneration. Key mechanistic insights reveal that MOTS-c orchestrates cytoprotection against TBHP-induced apoptosis and senescence via AMPK/SIRT1 pathway activation, effectively preserving ECM homeostasis by enhancing anabolism (collagen II/aggrecan) while suppressing fibrotic transformation (collagen I). The injectable hydrogel platform demonstrated translational potential in vivo, restoring disc biomechanical integrity (DHI% and MRI T2 signal) and mitigating histological degeneration through synergistic mitochondrial rejuvenation and structural support. This work advances IDD therapeutics by bridging mitochondrial medicine with functional biomaterials, offering a minimally invasive, biology-driven paradigm to address the unmet clinical need for disc regeneration. Future studies should explore dose optimization and long-term biocompatibility to accelerate the clinical translation of this mitochondria-hydrogel hybrid therapy.

## CRediT authorship contribution statement

**Yuan Lin:** Writing – original draft. **Hui Tao:** Writing – review & editing.

## Statement of significance

This study introduces a mitochondria-targeted therapeutic strategy for intervertebral disc degeneration (IDD) by integrating a mitochondrial-derived peptide (MOTS-c) with a self-assembling RADA16-I hydrogel (see Table 5).

Innovative Biomaterial Design: We engineered RAD/RMOTS-c, a functionalized hydrogel enabling sustained MOTS-c release while providing a three dimensional (3D) microenvironment for nucleus pulposus mesenchymal stem cell (NP-MSC) adhesion and extracellular matrix (ECM) regeneration. This dual-action platform addresses limitations of conventional therapies, such as rapid drug clearance and lack of structural repair.

Mechanistic Insight: MOTS-c mitigates oxidative stress-induced NP-MSC apoptosis and senescence by activating the AMPK/SIRT1 pathway, restoring mitochondrial homeostasis, and enhancing ECM synthesis (Collagen II↑, Collagen I↓). This is the first study to link MOTS-c's anti-degenerative effects to AMPK/SIRT1 signaling in NP-MSCs.

Translational Impact: In a rat IDD model, RAD/RMOTS-c hydrogel preserved disc height, restored T2-weighted MRI signals, and reversed ECM degradation, outperforming standalone RADA16-I. The results validated its potential as a minimally invasive, biology-driven therapy for IDD.

Broader Implications: Our work bridges mitochondrial medicine and biomaterial engineering, offering a blueprint for peptide-functionalized hydrogels in treating degenerative diseases characterized by oxidative stress and stem cell dysfunction.

This multidisciplinary approach aligns with Materials Today Bio's focus on translational biomaterials that synergize molecular insights with functional design to address unmet clinical needs.

## Ethics approval and consent to participate

Animal experiments were approved by Anhui Medical University (No.20200783; The applied basic research of dual-presenting Sa12b- and BMP7-motified functionalized self-assembling peptide hydrogels in the repair of intervertebral disc degeneration; 2020/03/01) and the animals were cared for in agreement with institutional ethics guidelines. All subjects signed informed consent forms and were approved by the Biomedical Ethics Committee of Anhui Medical University (NO.20200792).

## Consent for publication

Not applicable.

## Funding source

This work was supported by the Excellent Youth Scientific Research Project of Higher Education Institutions in Anhui Province (2022AH030117) , the Clinical and Translational Research Project of Anhui Province (202427b10020054) , the National Natural Science Foundation of China (No.82072492) and the Scientific Research Project in the Natural Science Category of Higher Education Institutions in Anhui Province (2023AH053307).

## Declaration of competing interest

The authors declare that they have no known competing financial interests or personal relationships that could have appeared to influence the work reported in this paper.

## Data Availability

Data will be made available on request.
